# Supplementary material for: Re-convolving the compositional landscape of primary and recurrent glioblastoma reveals prognostic and targetable tissue states
Source: Nat Commun. 2023 May 4;14:2586. doi: 10.1038/s41467-023-38186-1 (PMC10160047; doi:10.1038/s41467-023-38186-1)
Supplement: Supplementary file 1 — Supplementary info [file 41467_2023_38186_MOESM1_ESM.pdf]

Supplementary Results

Single nucleus RNAseq reveals proliferative, astrocyte-like/mesenchymal, and progenitor-like/proneural states in both primary and recurrent GBM

Radiographically, GBM typically has a CE core surrounded by a non-enhancing infiltrated brain that is highlighted by FLAIR-signal abnormality by MRI (**Supplementary Fig.1a**). The histopathological features of the resected tumor can vary from highly cellular tumor with vascular proliferation to less cellular infiltrated brain. These features are shown in **Supplementary Fig. 2h**, demonstrating samples with a cellular GBM core (red star in **Supplementary Fig. 1a, Supplementary Fig. 2h PA1, PA2, PA3, and PO2_1**) and others with overlying cortex (green star in **Supplementary Fig. 1a, Supplementary Fig. 2h PO2_2 and PO1**), which we use below.

To explore the heterogeneity of primary GBM, we analyzed several banked surgical samples using snRNAseq as shown in (**Supplementary Fig. 1a**). A total of 8 samples from 7 patients were selected for analysis (**Supplementary Dataset 1**). Neuropathological assessment of tumor cellularity ranged from cellular tumor with hallmarks of GBM, to reactive brain parenchyma with few atypical cells. This assessment was made on Hematoxylin and Eosin (H&E) stained formalin fixed paraffin embedded sections adjacent to or frozen cryosections of the frozen tissue analyzed by snRNAseq (**Supplementary Fig. 2h)**. We isolated nuclei from the frozen tissue and subjected them to snRNAseq followed by downstream analyses including clustering, differential gene expression analysis, cluster marker detection, and gene set enrichment analysis (GSEA) as outlined (**Supplementary Fig. 1a**). 15189 nuclei passed our QC (**Supplementary Dataset 1**). To distinguish putative glioma cells from non-neoplastic cells, we employed an established approach that infers large scale copy number alterations/variations (CNV) from RNA expression profiles [1]. Chromosomal heat maps showing putative neoplastic nuclei are shown in **Supplementary Fig. 2a-g**. Next, we also applied a second method to label nuclei based on a "malignancy score", which we have previously shown to be a robust metric to distinguish glioma cells from non-neoplastic cells [2,3], and the consensus nuclei designated by both methods was used for downstream analysis. Nuclei with no consensus CNV status were excluded (4.7%). Uniform manifold approximation and projection (UMAP) plots from individual cases labeled by transformation status are shown in **Supplementary Fig. 1b**. We identified 7954 putative neoplastic nuclei with inferred large scale chromosomal CNV (CNVpos/glioma nuclei). Glioma nuclei showed multiple chromosomal alterations including gains of chromosome 7 and losses of chromosome 10 (**Supplementary Fig. 2**). Having identified neoplastic and non-neoplastic nuclei, we aligned the datasets from multiple samples and performed clustering analyses separately on CNVpos (glioma) nuclei from all cases using shared nearest neighbor and the smart local moving algorithm [4]. A UMAP plot is shown for all primary glioma nuclei non-neoplastic nuclei color-coded by glioma state/lineage **Supplementary Fig. 1c**. This approach identified 6 distinct clusters: these resembled progenitors (oligodendrocyte-progenitors (gl_PN1 - proneural) and neural-progenitors (gl_PN2 - proneural), astrocytes (gl_Mes1 and gl_Mes2 - mesenchymal), and proliferative cells (gl_Pro1 and gl_Pro2).

The identity of the glioma states is akin to previously described glioma states, as demonstrated by the enrichment of several gene lists from [5-8] **– (Supplementary Fig. 1d, Supplementary Dataset 2)**. For example, gl_Pro1 and gl_Pro2 showed enrichment in gene sets specific for cell-cycle phases [8], with gl_Pro1 showing highest enrichment of G2/M genes (Gobin_G1) and gl_Pro2 showing highest enrichment of G1/S phase genes and DNA repair related genes (Gobin_G3). Clusters gl_PN1 showed enrichment of the Verhaak's proneural, and

OPC signature genes, while gl_PN2 showed enrichment of NPC signature genes. Finally, gl_Mes1 showed enrichment of astrocyte-like signatures and Verhaak's classical signature while cluster gl_Mes2 showed enrichment of several gene sets related to reactive astrocytes, and Verhaak's mesenchymal signature [5,7,9]. Our clustering is consistent with that described in Neftel et al. 2019 [6] and Wang et al. 2019 [9], and the states we describe are compatible with those in Yuan et al 2018 [3]. To further clarify the cellular phenotypes represented in our glioma clusters, we measured the enrichment of the major biologic process and molecular function gene ontologies (GO) in the glioma state top gene markers (see methods)**.** GO enrichment analysis demonstrated enrichment of GO's relating to locomotion, neurogenesis, neuronal migration, and cell projection in gl_PN1 markers genes; Notch signaling, neuron development, and GABA reuptake differentiation, and synaptic signaling in gl_PN2 genes; response to organic substances, ion homeostasis, and signaling by tyrosine kinases in gl_Mes1 genes; response to cytokines, interferon gamma, and leukocyte activation and immune response in gl_Mes2 genes; mitosis and nuclear division in gl_Pro1, and S-phase, DNA replication, and DNA repair in gl_Pro2(**Supplementary Fig. 1f** and **Supplementary Dataset 2**). The identities of the clusters can also be appreciated by examining select gene markers **Supplementary Fig. 1e and Supplementary Dataset 2.** gl_Pro1 expressed cell-cycle genes *TOP2A*, *CENPF*, and *AURKB*. gl_Pro2 showed highest expression of DNA damage/repair including *FANCI*, *HELLS*, and *XRCC2*. gl_PN2 showed high levels of *CD24*, *MEG3*, and *SOX4*. gl_Mes1 showed high levels of protoplasmic astrocyte genes including *SLC1A3, LIFR, ATP1A2, C1orf61*, and *NTM*, while gl_Mes2 showed highest expression levels for reactive astrocyte and immune genes including *CLU, VIM*, and *SAT1*. While our glioma states resemble those described in the literature, less is known about whether glioma cells assume similar states in the recurrent setting. Therefore, we bridged this gap by directly analyzing recurrent IDH-WT glioma samples using the same approach we used for primary GBM samples.

To define the states of IDH-WT glioma in the post-treatment recurrence setting, we analyzed 8 cases of post-recurrent IDH-WT glioma using snRNAseq (**Supplementary Fig. 3a**). We identified 8908 neoplastic nuclei harboring large-scale CNV (**Supplementary Fig. 4**). Of the eight cases, two were paired recurrences from the primary samples (TB5124 – recurrent of TB4916, and TB5053 – recurrent of TB4718, see respective section on comparing paired samples below). We treated recurrent gliomas similarly to the treatment naïve primary tumors and clustered all neoplastic nuclei together. Like primary gliomas, we found that recurrent glioma clusters can be assigned two proneural, two mesenchymal, and two proliferative states (**Supplementary Fig. 3b**). The gene markers of the recurrent glioma states are enriched for similar ontologies to those seen for primary glioma states (**Supplementary Fig. 3c** and **Supplementary Dataset 3**), showed similar patterns of enrichment for the previously presented gene sets in **Supplementary Fig. 1d** (**Supplementary Fig. 3d**), and displayed comparable gene marker expression (**Supplementary Fig. 3f**). These results demonstrate that post-treatment recurrent glioma states closely resemble states observed in the primary pre-treatment setting. Indeed, Pearson correlation analysis demonstrates that corresponding states were positively correlated (**Supplementary Fig. 3e**). The correlation patterns reveal that gl_Mes1 and gl_Mes2 are positively correlated with each other in the primary and recurrent settings. This is also seen with gl_PN1 and gl_PN2, as well as gl_Pro1 and gl_Pro2. We therefore contend that a view of primary and recurrent glioma states may benefit from simplification and embrace a viewpoint that primary and recurrent glioma states can be classified as progenitor-like/proneural (gl_PN1 and gl_PN2), astrocyte-like/mesenchymal (gl_Mes1 and gl_Mes2), and proliferative (gl_Pro2 and

gl_Pro1) states. A select set of markers of both primary and recurrent GBM states is provided in **Supplementary Fig. 4i**. Assigning cell cycle scores using Seurat cell-cycle score assignment reveals that gl_Pro1 has the majority of cells in G2M phase, whilst gl_Pro2 has the majority of cells in S phase **Supplementary Fig. 4i**. Integration of both primary and recurrent glioma nuclei shows cells from primary and recurrent samples overlap in the UMAP space, and that this overlap is seen for all 6 GBM states (**Supplementary Fig. 5g**).

While the transcriptional signatures of glioma are relatively well defined, the spatial distribution of these glioma states is less well understood. Given the marked difference in cellular composition between the cortex and the deeper (typically more heavily infiltrated) white matter, and the highly cellular tumor core, we asked if these different anatomic regions harbor distinct glioma states. In other words, we posited that the cellular microenvironment of glioma influences glioma states. Specifically, we hypothesized that we would find more glioma cells that resemble astrocytes (astrocyte-like/mesenchymal glioma) or neurons (progenitor-like - specifically gl_PN2) in the cortical margins. To address this question, we examined the expression of select combinations of glioma state transcripts using in situ hybridization (ISH) across the cellular tumor and the infiltrated cortical margin. We used probes to detect PTPRZ1 (high in glioma), CLU (high in astrocytes and astrocyte-like/mesenchymal glioma), SOX2 (high glioma), NOVA1 (high in progenitor-like/proneural glioma), and MEG3 (high in neurons and progenitor-like/proneural glioma - gl_PN2) in the cellular core and overlying infiltrated cortical margin in 5 cases of primary GBM (**Supplementary Fig. 5a, c**). We found that a significantly higher proportion of PTPRZ1+ glioma cells co-expressed CLU (high in gl_Mes2) in the cortex versus the core (**Supplementary Fig. 5b**). Similarly, we found a significantly higher proportion of SOX2+NOVA1+MEG3+ glioma cells in the cortex versus the core (**Supplementary Fig. 5d**). These findings indicate that the different glioma states have distinct distributions throughout the landscape of glioma and suggest that local tissue cellular composition and perhaps other microenvironmental influences can affect glioma states. We note that astrocyte-like/mesenchymal glioma states were negatively correlated with proliferative states. Consistent with this result, our ISH findings demonstrated a significantly smaller proportion of CLU+ cells that co-expressed TOP2A (mean=31.71388837%, Standard deviation = 15.73850618, one-tailed t-test p= 0.000249641, n=5, **Supplementary Fig. 5e-f**).

Comparison between primary and recurrent glioma pairs

Not surprisingly, the recurrent tumors did not show identical chromosomal CNVs with their primary counterparts. While TB5014 retained the CNV of TB4916 (gain of 7, loss of 10 and 14) and acquired additional alterations including gains in chromosomes 19 and 20 (**Supplementary Fig. 2f and 4a)**, TB5053 showed a complex gains and losses across multiple chromosomes (**Supplementary Fig. 2g and 4b)**.

In the main text, we note that gl_PN1 is depleted from our recurrent GBM samples (**Figure 4a**). This is consistent with the literature [10], since the Verhaak classical subtype resembles our gl_PN1, which showed positive enrichment scores of the Verhaak's classical gene set. Of the non-neoplastic cell types, OPCs were depleted in recurrence. This may be explained by the fact the OPCs are the proliferative cell type in the brain and glioma treatment with chemotherapy and radiotherapy depletes proliferative cells, as have been previously demonstrated [11].

Analysis of low-grade glioma and epilepsy cases

To sample states of myeloid cells and astrocytes across different disease states, we chose to analyze the microenvironment of low-grade glioma (LGG) and temporal lobe epilepsy. We conducted snRNAseq on 6 cases: two IDH-mutant oligodendroglioma (TB3652 & TB3926), one

IDH-mutant astrocytoma (TB4100), and three temporal lobe epilepsies (TB4189, TB4437, & TB4957). We identified 970, 1154, 1036 nuclei for LGG cases TB3652, TB3926, and TB4100, respectively. We identified CNVpos nuclei using a combination of chromosomal CNV, clustering, and tumor marker expression as shown in **Supplementary Fig. 6**. Cases TB3652 and TB3926 had typical chromosome 1p and 19q codeletions (**Supplementary Fig. 6a, d**), and harbored 817 and 942 CNVpos nuclei, respectively (**Supplementary Fig. 6b, e**). The tumor nuclei expressed tumor markers *SOX2, EGFR*, and *PTPRZ1*, and/or OPC markers *DSCAM* and *TNR*; myeloid cells expressed *CD74*, *C3*, *ITGAX/CD11c*, *ITM2B*, and/or *HLA-B*; while oligodendrocytes expressed *MBP* and *MOG* (**Supplementary Fig. 6c, f**). 382 CNVpos nuclei were found in case TB4100, which did not harbor CNVs across most cells, and CNVpos nuclei were identified by clustering and marker expression as noted above. Of the epilepsy cases, we identified 2558, 179, and 138 nuclei in cases TB4189, TB4437, and TB4957, respectively. **Supplementary Fig. 7a-c** show marker expression in cases TB4437, TB4189, and TB4957, where markers of astrocytes (*GFAP, AQP4, SLC1A2, SLC1A3*), neurons (*RBFOX3, MEG3, GAD1,* and *SLC17A6*), myeloid cells (*CD74, ITGAX, C3, ITM2B*), oligodendrocytes (*MBP, MOG, OPALIN,* and *CNP*), and OPCs (*DSCAM, TNR, SOX2,* and *PDGFRA*). The CNVneg nuclei from all LGG and epilepsy cases were combined with those from primary and recurrent IDH-WT GBM and were analyzed as presented in the section below (myeloid cells) and main text (astrocytes).

Astrocytes cluster into three distinct astrocyte cell states

Based on the resemblance to known astrocyte phenotypes we curated three gene sets (**Supplementary Dataset 4**), which represent three major astrocyte states (protoplasmic, reactive-1, and reactive-2), and then clustered astrocyte nuclei using Ward D2 hierarchical clustering on the Manhattan distance of the enrichment scores (overlaid on the 3D tSNE plots in Figure 2, into a protoplasmic cluster (Ast1), and two reactive clusters (Ast2 and Ast3 – as described in the main text (**Fig. 2a**). We asked whether our method of clustering astrocytes, described in figure 2, can result in similar clusters to more "unbiased" methods. Thus, we performed Louvain clustering on shared nearest neighbor graphs (created through igraph – k=500 – **Supplementary Fig. 13a**). Examination of marker expression for each cluster demonstrate that Louv 2 is similar to Ast1 - with expression of protoplasmic genes, Louv 3 is similar to Ast2 – with expression of *PLP1* and ribosomal genes, and Louv 1 is similar to Ast3 – with expression of *C3* and *CD44* (**Supplementary Fig. 13b**). Examination of the overlap of astrocyte calls between the method employed in figure 2 and the "unbiased" clustering reveals that the unbiased Louv clusters overlap to high extent with those described in Figure 1, as described above (**Supplementary Fig. 13c**). Therefore, overall, we conclude that the clustering approach we employed in figure 2 is highly analogous to unbiased clustering. However, there were some differences in the transcriptional features of the unsupervised clusters when compared to the clusters we generated through supervised classification (Ast1-Ast3). While most markers for Ast1-Ast3 were enriched in one unsupervised cluster, *CLU*, a marker for Ast3, is expressed at similar levels in two of the unsupervised clusters (Louv1 and Louv 3).

Analysis of myeloid cell states

Myeloid cells have been implicated in modulating glioma migration, infiltration, and progression [12]. We identified 5925 nuclei we classified as myeloid cells. Unbiased clustering revealed 8 subclusters which we then used to assign the specific myeloid lineages. We merged clusters with similar enrichment scores of gene sets representing microglia-derived tumor-associated macrophages (mgTAM), monocyte-derived TAMs (moTAM), proliferative TAMs (prTAMs), and T-cells as described in [13] - Myeloid subclusters 0 and 9 were combined as Myel1 (baseline),

subclusters 1 and 3 – moTAM (monocyte derived TAMs); subclusters 2, 4, 6, and 8 as mgTAM (microglia-derived TAMs); subcluster 5 was kept as prTAM (proliferative TAMs); and subcluster 7 was kept as T-cells. . The enrichment of these gene sets in the final myeloid states is provided in **Supplementary Fig. 9e**. A subset of myeloid cells showed mixed enrichment scores across mgTAM, moTAM, and dendritic cells, and were considered baseline (referred to as Myel1). Overall, we classified 2678, 1346, 1364, 360, and 177 nuclei as Myel1, moTAM, mgTAM, prTAM, and T-cells, respectively, and these are shown in 3D tSNE space in **Supplementary Fig. 9a**. Myel1 state showed higher expression of *SAT1, CEBPD*, and *GLUL* (**Supplementary Fig. 9c**-top row). moTAM showed highest expression of *CD163, MS4A4E, NHSL1, FMN1,* and *MSR1* (**Supplementary Fig. 9c**-2nd row). mgTAM showed highest expression of *SORL1, RIN3, ITGAX, HS3ST4,* and *FRMD4A* (**Supplementary Fig. 9c**-3rd row). prTAMs showed highest expression of *CST3, MEF2A, DBI, PLXDC2,* and *DOCK4* (**Supplementary Fig. 9c**-4th row). Finally, T-cells showed highest expression of *CD2, CD247, CD96, FYN,* and *SKAP1* (**Supplementary Fig. 9c**-5th row). Different myeloid states were accounted for different conditions (**Supplementary Fig. 9b).** While Myel1 was present in Epilepsy, primary and recurrent GBM, mgTAM was the main state found in LGG, but was also in primary and recurrent GBM. moTAM, T-cells, and prTAM were found in primarily in recurrent GBM (**Supplementary Fig. 9d)**. The gene-wise DGE between myeloid states and the myeloid state markers are provided in **Supplementary Dataset 5**.

snRNAseq of samples used for ST – a validation dataset

Single nuclei from each ST patient were analyzed when available (n = 7). The nuclei were obtained, cleaned, and analyzed as described elsewhere. CNVpos nuclei were identified using inferCNV (**Supplementary Fig. 8a-g,** sample QC, number of nuclei per sample as well as lineage assignment is provided in **Supplementary Dataset 1).** They were classified as was done for the previously presented datasets. The CNVneg nuclei were then classified into cell types using the *singleR* package (de.method="wilcox") with the previously annotated single nuclei data set as a reference[14]. Proportions of nuclei per cell type are included in **Supplemental dataset-1.** Using the compositional matrix of these samples, they were able to be classified into tissue states using k-means clustering with the centers of the discovery data set samples supplied as centers (**Supplementary Fig. 8h**). The integrated CNVneg nuclei are shown in a UMAP (**Supplementary Fig. 8i**).

Spatial cross-correlation analysis of deconvolved cell type proportions

Our analysis of cell type composition in snRNAseq samples highlights prognostically relevant compositional patterns. To examine these patterns with spatial resolution, we analyzed 9 IDH-WT GBM samples using spatial transcriptomics and deconvolution (see methods). To validate the accuracy of our deconvolution, we compared the distribution of deconvolved cell type proportions to fluorescent staining for select cell type markers. **Supplementary Fig. 11a-b** show the deconvolved proportion of neurons in a subset of ST samples alongside fluorescent staining of the same samples for NeuN, a canonical marker for neurons – see **Supplementary Fig. 10b**. This highlights that our deconvolution approach was able to reflect patterns of spatial heterogeneity that were also suggested by fluorescent staining. **Supplementary Fig. 11c-d** shows deconvolved proportions of select cell types in 2 ST samples and shows that cell types whose proportions covary in each tissue-state show similar patterns of spatial heterogeneity to each other across multiple samples. We quantified and aggregated trends across our 9 ST samples using spatial cross-correlation and tested them for significance—see methods for details. To determine the relative representation of tumor within each ST sample and confirm the ability of our deconvolution approach to identify tumor, we correlated nuclear density (cellularity)

obtained from immunohistochemical staining for DAPI (**Supplementary Fig. 10a**) with our deconvolved cell type proportions. BayesSpace was used to segment each sample into clusters containing transcriptionally similar spots (see methods). A total of 33 clusters were generated (**Supplementary Fig. 10d)**. The density of nuclei was obtained for all of these clusters across the data set, and we calculated the correlation with the deconvolved proportion of each cell type (**Supplementary Dataset 1**). The total proportion of CNVpos cell types was positively and significantly correlated with density of nuclei (correlation: 0.388, p=0.025). **Supplementary Fig. 11e** shows a representative sample with DAPI staining and **Supplementary Fig. 11f** shows the same sample segmented by BayesSpace generated clusters and colored by the proportion of deconvolved CNVpos cell types present in that cluster.

The spatial landscape of glioma associated tissue-states in primary and recurrent GBM

To understand the spatial landscape of primary and recurrent glioma, we mapped the distribution of our "tissue-state" signatures in space in primary and recurrent GBM. First, we tested one of our cases that we utilized for snRNAseq (PO2) and took 48 localized biopsies that we analyzed using plate-seq [15]. Immunofluorescence of frozen sections taken prior to analysis revealed a cellular DAPI-dense glioma core and a NeuN rich cortical margin (**Supplementary Fig. 12a**). We conducted GSEA analysis of our tissue-state signatures in the RNAseq data from the localized biopsies and mapped that against the location of the biopsies (**Supplementary Fig. 12b**). Tissue-state C signature was highest in the core, compared to tissue-state A signature, which was highest in the cortical margin. Tissue-state B signature showed a patchier distribution with foci of enrichment in both the core and margin. Interestingly, the intermediate region between the core and cortex, showed mixed enrichment across all three tissue-states. This data highlights the anatomic localization of tissue-state signatures and underscores the heterogeneous patterns in the intermediate non-cortical "margin" region.

Next, we performed deconvolution on a previously published dataset of bulk RNA sequencing from MRI-localized biopsies of primary and recurrent GBM, and control brain samples [7] to assess the abundance of neoplastic and non-neoplastic cell types in different radiographic regions of primary and recurrent GBM. Our results showed that in contrast enhancing regions of primary GBM, the cell types associated with tissue states B and C were more abundant than cell types associated with tissue state A, while in the contrast enhancing regions of recurrent GBM, the cell types associated with tissue state B were more abundant than cell types associated with tissue state C or A. The FLAIR+ samples in primary GBM showed a mixture of neoplastic and non-neoplastic cell types from all three tissue states, while the FLAIR+ samples from recurrent GBM showed predominantly non-neoplastic cells, with highest abundance of cell types of tissue state A. As expected, control samples were also predominately composed of non-neoplastic cell types associated with tissue state A. (**Supplementary Fig. 12c)**. We also assessed for the expression of tissue-state signature genes in these same samples. This analysis showed similar patterns to those of the deconvolved cell types (**Supplementary Fig. 12c**). In summary, contrast enhancing regions in both primary and recurrent tumors predominantly represent neoplastic and reactive cell types, but the distribution of specific glioma subtypes varies between primary and recurrent tissue. Non-enhancing margins of recurrent GBM samples predominantly represent reactive/gliotic brain tissue with relatively low levels of tumor infiltration, whereas the non-enhancing margins of primary GBM can contain a wider range of pathological features, including regions of abundant glioma infiltration.

Astrocyte CLU alters U87 glioma cell gene expression

In examining the cellular milieu co-inhabiting tissue state B, we focused on Ast3, an astrocytic state with high expression of Clusterin (CLU). Astrocytic CLU is known to reduce amyloid accumulation in mouse models of Alzheimer's disease and is thought to be neuroprotective[16-18]. CLU is upregulated in GBM and can protect GBM cells from radiation-induced apoptosis [19]. However, little is known about the interaction between CLU+ astrocytes (i.e. Ast3) and GBM. We first identified the genes that were significantly correlated with *CLU* expression (using the psych::corr.test R function) in astrocytes that have high *CLU* expression, defined as in the 3rd and 4th quantiles of normalized *CLU* levels. These include *ATP1B2, F3, AQP4, GJA1, CHI3L2, CHI3L1, LGALS1,* and *LGALS3* among others (**Supplementary Fig. 13d** and **Supplementary Dataset 8**). Analysis of pathways enriched in *CLU*-correlated genes reveals they encompass Reactome and KEGG pathways involved in signal transduction, Rho GTPases, Hippo signaling, and translation (**Supplementary Fig. 13e**). With a testable Ast3 (*CLU*-high) astrocytic signature at hand, we modeled an Ast3-like astrocyte state *in vitro* by overexpressing *CLU* in human astrocytes (**Supplementary Fig. 13f**). As a separate experimental condition we overexpressed *LGALS3*. rt-qPCR analysis shows that merely co-culturing astrocytes with U87 glioma cells leads to reduction of astrocytic *SOX2, NES, CLU,* and *HES5* expression. Rt-qPCR confirms *CLU* and *LGALS3* overexpression in *CLU*- and *LGALS3*- astrocytes, respectively, and reveals *CLU* astrocytes increase *HES5* expression, whereas *LGALS3 astrocytes* increase *NES* expression, both when compared to GFP control astrocytes in the setting of U87 co-culture (**Supplementary Fig. 13j**). CHI3L1, and Ast3 gene, was increased in both *LGALS3*+ and *CLU*+ astrocytes. Since both *CLU*+ and *LGALS3*+ astrocytes model some aspects of Ast3, and only *CLU*+ astrocytes significantly increased astrocytic *CLU*, we chose to use the *CLU*+ astrocytes as an Ast3-like model and analyzed those cells further. Comparing the genes differentially expressed between sorted *CLU*-overexpressing versus control astrocytes revealed 274 differentially expressed genes, including many that are positively correlated with *CLU* levels as defined by human glioma-associated astrocytes (**Supplementary Fig. 13g** and **Supplementary Dataset 8**). These genes are enriched in KEGG/Reactome pathways that encompass Hippo signaling, and extracellular matrix organization (**Supplementary Fig. 13h**). Notably, these *CLU* induced genes are significantly positively enriched in the Ast3 gene signature (**Supplementary Fig. 13i**). These results provide support for the resemblance between Ast3 cells and *CLU*+ astrocytes. Next, we focused on glioma cells and asked if co-culture of U87 glioma cells with astrocytes leads to altered glioma gene expression. rt-qPCR of sorted U87 glioma showed that merely co-culturing U87 glioma with astrocytes leads to increased *SOX2* and *HES1* expression. When co-cultured with CLU astrocytes *HES5* is increased in U87 cells, whereas *HES1* is reduced in U87 cells co-cultured with *LGALS3* astrocytes (**Supplementary Fig. 13k)**. RNAseq of U87 glioma co-cultured with control (*GFP*) astrocytes leads to enrichment of gene ontologies involved in monocyte differentiation and leukocyte migration (**Supplementary Fig. 13l and Supplementary Dataset 8.** When co-cultured with CLU-astrocytes, the transcriptome of U87 glioma cells is enriched in ontologies involved in glial differentiation, neural precursor proliferation, and biosynthesis of unsaturated fatty acids (**Supplementary Fig. 13m and Supplementary Dataset 8).** Together, these results show that astrocytes can exert different effects on glioma gene expression, and Ast3-like astrocytes promote a signature related to glial differentiation and precursor proliferation.

Supplementary figures

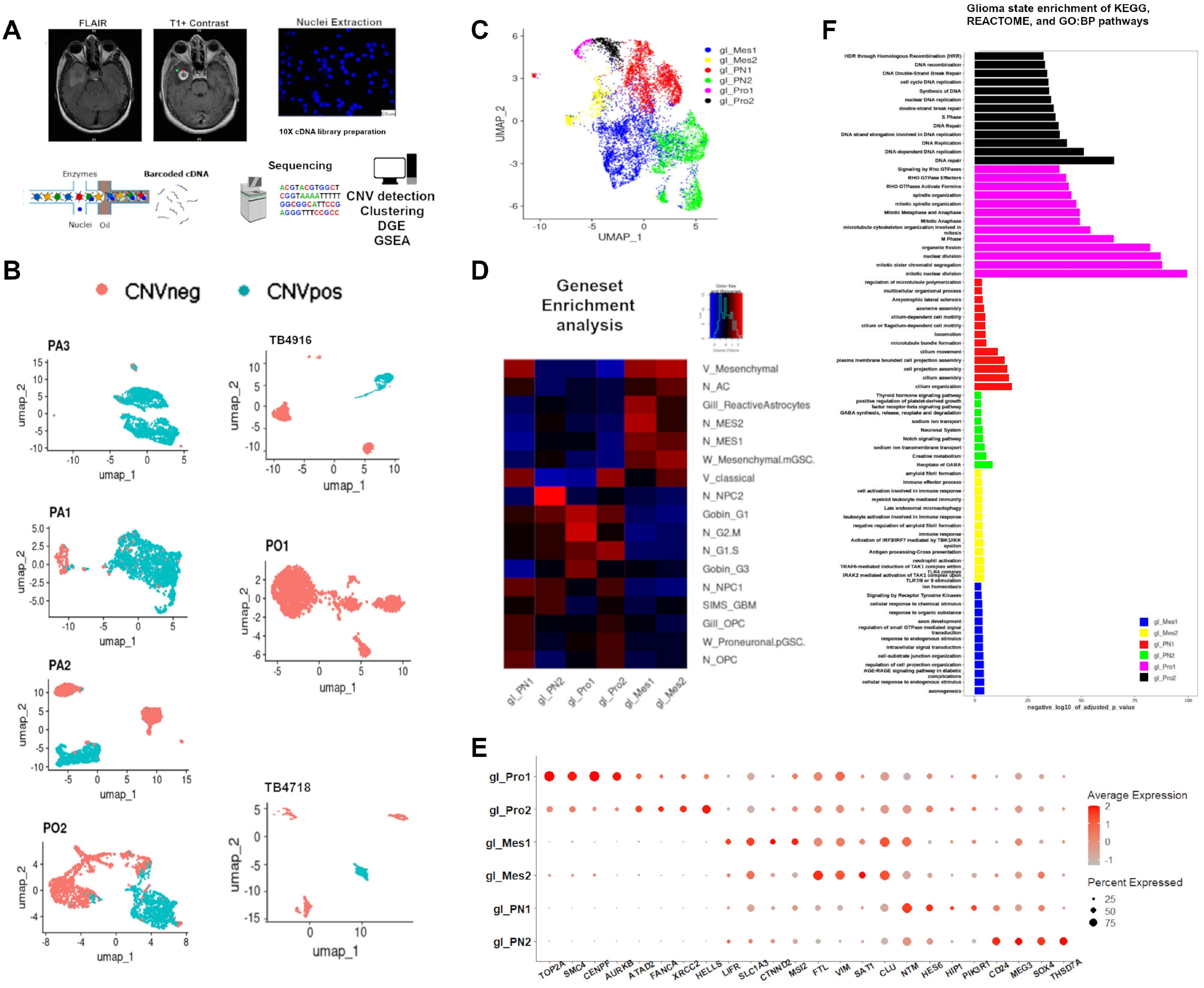

A
FLAIR
T1+ Contrast
Nuclei Extraction
10X cDNA library preparation
Enzymes
Barcoded cDNA
Nuclei
Oil
Sequencing
ACGTACGTGGCT
CGGTAAAATTTTT
GGCGGCATTCCG
AGGGTTTCCGCC
CNV detection
Clustering
DGE
GSEA
B
CNVneg
CNVpos
PA3
TB4916
PA1
PO1
PA2
PO2
TB4718
umap_1
umap_2
C
UMAP_1
UMAP_2
gl_Mes1
gl_Mes2
gl_PN1
gl_PN2
gl_Pro1
gl_Pro2
D
Geneset Enrichment analysis
V_Mesenchymal
N_AC
Gill_ReactiveAstrocytes
N_MES2
N_MES1
W_Mesenchymal.mGSC.
V_classical
N_NPC2
Gobin_G1
N_G2.M
N_G1.S
Gobin_G3
N_NPC1
SIMS_GBM
Gill_OPC
W_Proneuronal.pGSC.
N_OPC
gl_PN1
gl_PN2
gl_Pro1
gl_Pro2
gl_Mes1
gl_Mes2
E
gl_Pro1
gl_Pro2
gl_Mes1
gl_Mes2
gl_PN1
gl_PN2
TOP2A
SMC4
CENPF
AURKB
ATAD2
FANCA
XRCC2
HELLS
LIFR
SLC1A3
CTNND2
MSI2
FTL
VIM
SAT1
CLU
NTM
HES6
HIP1
PIK3R1
CD24
MEG3
SOX4
THSD7A
Average Expression
Percent Expressed
F
Glioma state enrichment of KEGG, REACTOME, and GO:BP pathways
HDR through Homologous Recombination (HRR)
DNA recombination
DNA Double-Strand Break Repair
cell cycle DNA replication
Synthesis of DNA
nuclear DNA replication
double-strand break repair
S Phase
DNA Repair
DNA strand elongation involved in DNA replication
DNA Replication
DNA-dependent DNA replication
DNA repair
Signaling by Rho GTPases
RHO GTPase Effectors
RHO GTPases Activate Formins
spindle organization
mitotic spindle organization
Mitotic Metaphase and Anaphase
Mitotic Anaphase
microtubule cytoskeleton organization involved in mitosis
M Phase
organelle fission
nuclear division
mitotic sister chromatid segregation
mitotic nuclear division
regulation of microtubule polymerization
multicellular organismal process
Amyotrophic lateral sclerosis
axoneme assembly
cilium-dependent cell motility
cilium or flagellum-dependent cell motility
locomotion
microtubule bundle formation
cilium movement
plasma membrane bounded cell projection assembly
cell projection assembly
cilium assembly
cilium organization
Thyroid hormone signaling pathway
positive regulation of platelet-derived growth factor receptor-beta signaling pathway
GABA synthesis, release, reuptake and degradation
sodium ion transport
Neuronal System
Notch signaling pathway
sodium ion transmembrane transport
Creatine metabolism
Reuptake of GABA
amyloid fibril formation
immune effector process
cell activation involved in immune response
myeloid leukocyte mediated immunity
Late endosomal microautophagy
leukocyte activation involved in immune response
negative regulation of amyloid fibril formation
immune response
Activation of IRF3/IRF7 mediated by TBK1/IKK epsilon
Antigen processing-Cross presentation
neutrophil activation
ion homeostasis
Signaling by Receptor Tyrosine Kinases
cellular response to chemical stimulus
response to organic substance
axon development
regulation of small GTPase mediated signal transduction
response to endogenous stimulus
intracellular signal transduction
cell-substrate junction organization
regulation of cell projection organization
AGE-RAGE signaling pathway in diabetic complications
cellular response to endogenous stimulus
axonogenesis
negative_log10_of_adjusted_p_value

Supplementary Figure 1: snRNAseq-derived transcriptional states of putative neoplastic nuclei from primary IDH-wildtype GBM samples

a) Outline of Analytic Design: T 2/FLAIR and post-contrast T1 MRI sequences of a glioblastoma showing the classic radiological appearance of a glioblastoma (Case PO2); with a ring enhancing mass (red star) with surrounding increased FLAIR signal (green star). The tumor was resected and banked (frozen). Nuclei are extracted from frozen tissue and are subjected to droplet based single nuclei RNA sequencing using the 10X chromium platform. The resultant barcoded cDNA is then sequenced and analyzed. Analyses performed include identification of putative neoplastic cells by identifying cells with inferred copy number variations (CNV), clustering, differential gene expression (DGE), and gene set enrichment analysis (GSEA). Scale bars = 50 um. This panel was created with BioRender.com. **b)** Uniform-manifold approximation and projection (UMAP) graphs showing putative neoplastic (CNVpos) and non-neoplastic (CNVneg) nuclei from the seven primary IDH-wildtype glioma cases selected for analysis indicated by subpanels b1-b7. **c**) UMAP plot showing all putative CNVpos (**c**) nuclei from the seven primary glioma cases aligned and projected in shared UMAP spaces. The nuclei are color-coded by glioma state: Oligodendrocyte-progenitor-like (proneural - gl_PN1), Neural-progenitor-like (proneural - gl_PN2), Mesenchymal/astrocyte like (gl_Mes1 and gl_Mes2), and proliferative (gl_Pro1 & gl_Pro2). **d**) Geneset enrichment analysis (GSEA) of selected genesets from Verhaak et al. 2009 (v), Gobin M et al 2019, Gill et al 2014, Wang et al. 2019 (W), and Neftel et al. 2019 (N) showing enrichment of genes specific for states described in the literature in our described glioma states. **e**) Dot plots showing expression of certain markers in glioma states. **f**) Gene ontology (GO) term enrichment analysis (KEGG and REACTOME pathways and biological process GO) of the major terms enriched in glioma state top gene markers. The bars represent the negative log10 of the Bonferroni adjusted p.value, and are color-coded as in **c**.

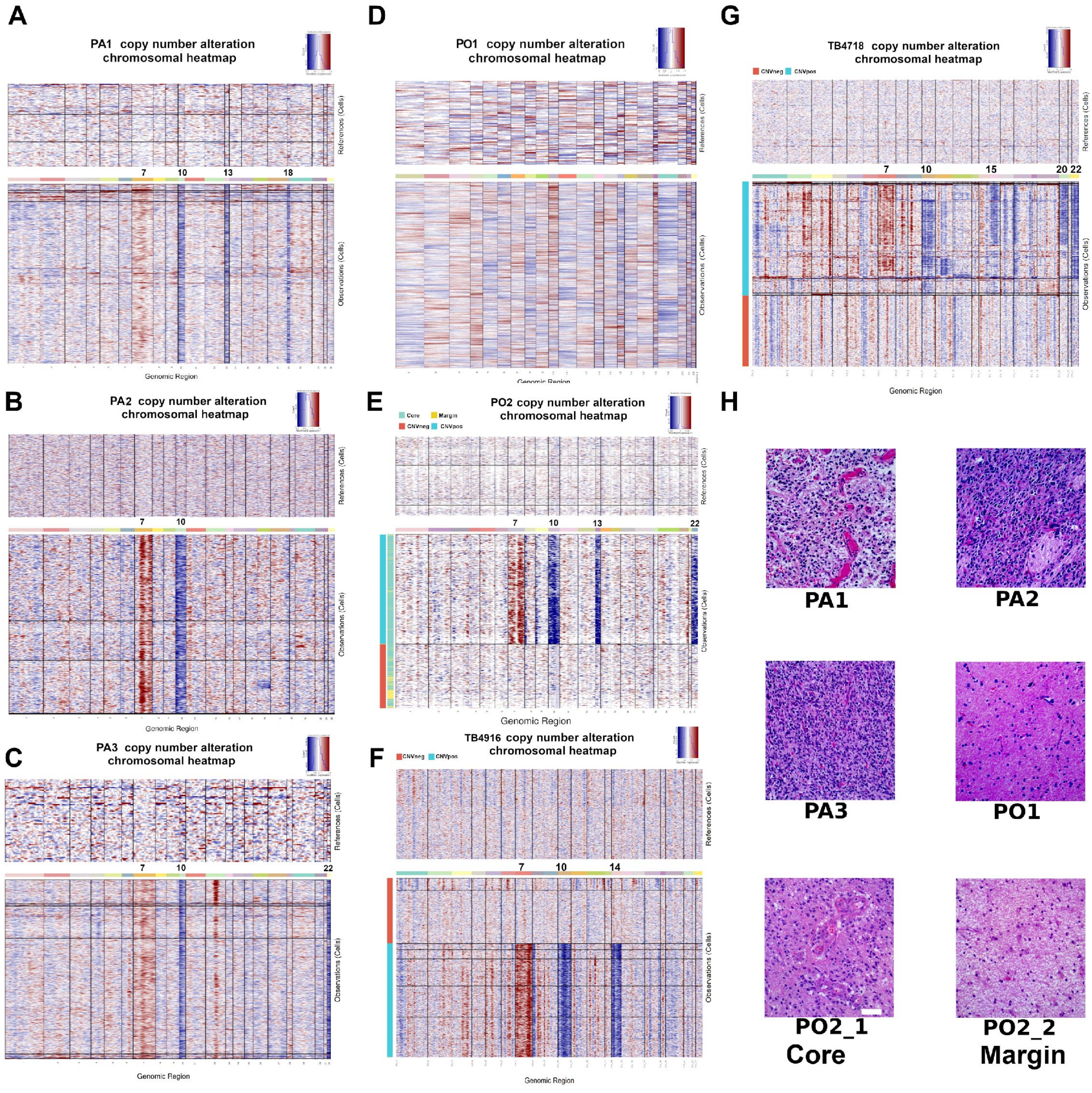

A
PA1 copy number alteration chromosomal heatmap
7 10 13 18
References (Cells)
Observations (Cells)
Genomic Region
B
PA2 copy number alteration chromosomal heatmap
7 10
References (Cells)
Observations (Cells)
Genomic Region
C
PA3 copy number alteration chromosomal heatmap
7 10 22
References (Cells)
Observations (Cells)
Genomic Region
D
PO1 copy number alteration chromosomal heatmap
References (Cells)
Observations (Cells)
Genomic Region
E
Core Margin
CNVneg CNVpos
PO2 copy number alteration chromosomal heatmap
7 10 13 22
References (Cells)
Observations (Cells)
Genomic Region
F
CNVneg CNVpos
TB4916 copy number alteration chromosomal heatmap
7 10 14
References (Cells)
Observations (Cells)
Genomic Region
G
CNVneg CNVpos
TB4718 copy number alteration chromosomal heatmap
7 10 15 20 22
References (Cells)
Observations (Cells)
Genomic Region
H
PA1
PA2
PA3
PO1
PO2_1 Core
PO2_2 Margin

Supplementary Figure 2: Identifying neoplastic nuclei based on chromosomal copy number alterations, and histopathologic characterization of glioma cases

a-g) Large scale chromosomal copy number alterations were inferred from RNA expression using InferCNV R package (see methods for details). The heat maps show gains (red) and losses (blue) in case PA1 (**a**), PA2 (**b**), PA3 **(c),** PO1 (**d**), PO2 (two samples – core and margin) **(e),** TB4916 **(f),** and TB4718 (**g**). **h)** Representative Hematoxylin and Eosin-stained section of the brain tissue used for single nuclei RNAseq of the first five cases (scale bar equals 50μm) . Some cases showed clear infiltration with glioma cells PA1, PA2, PA3, and PO2_c, PO2_2. Cases PO1 and PO2_m showed no clear evidence of cellular tumor.

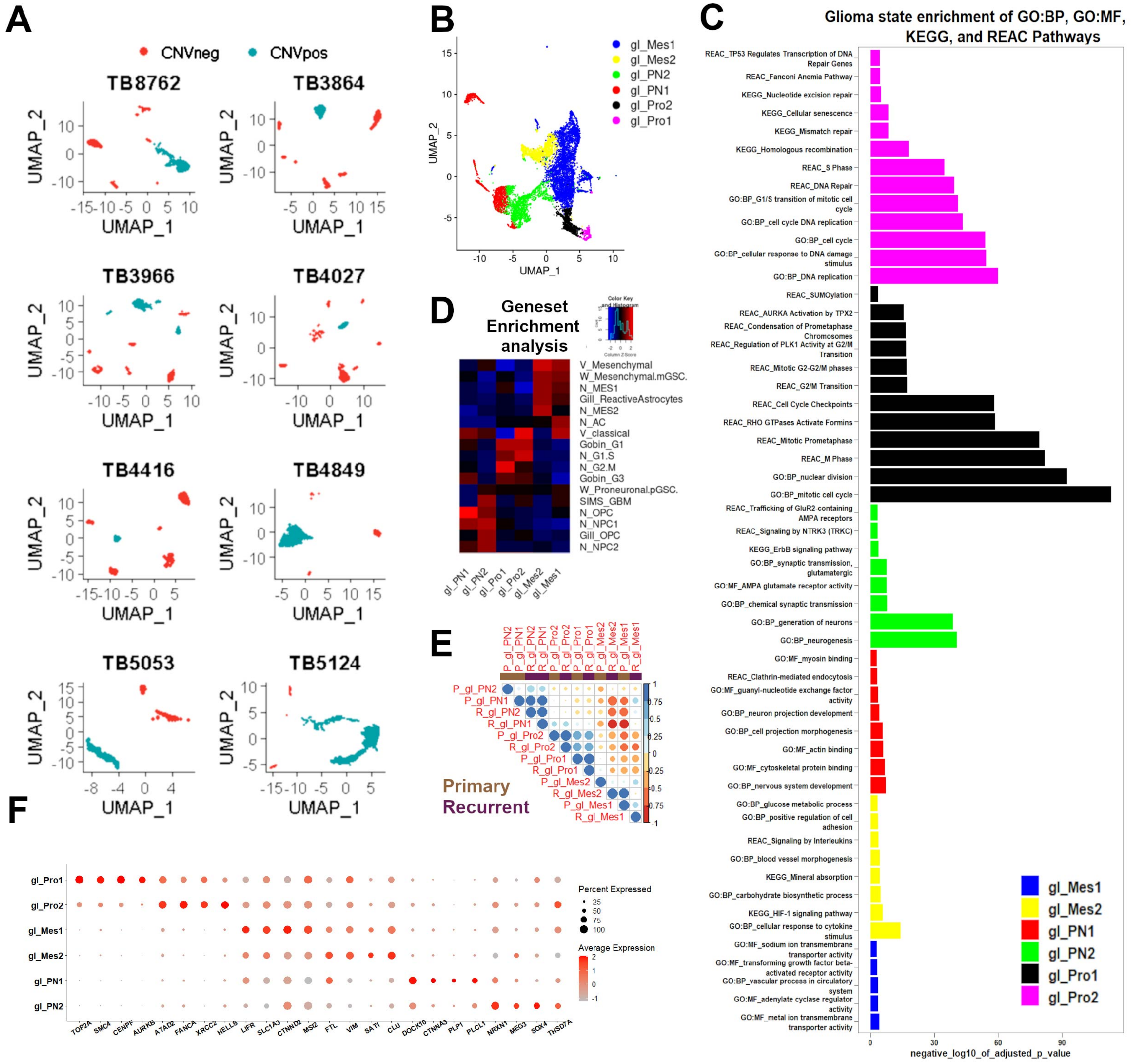

A
CNVneg
CNVpos
TB8762
TB3864
TB3966
TB4027
TB4416
TB4849
TB5053
TB5124
UMAP_1
UMAP_2
B
gl_Mes1
gl_Mes2
gl_PN2
gl_PN1
gl_Pro2
gl_Pro1
C
Glioma state enrichment of GO:BP, GO:MF, KEGG, and REAC Pathways
REAC_TP53 Regulates Transcription of DNA Repair Genes
REAC_Fanconi Anemia Pathway
KEGG_Nucleotide excision repair
KEGG_Cellular senescence
KEGG_Mismatch repair
KEGG_Homologous recombination
REAC_S Phase
REAC_DNA Repair
GO:BP_G1/S transition of mitotic cell cycle
GO:BP_cell cycle DNA replication
GO:BP_cell cycle
GO:BP_cellular response to DNA damage stimulus
GO:BP_DNA replication
REAC_SUMOylation
REAC_AURKA Activation by TPX2
REAC_Condensation of Prometaphase Chromosomes
REAC_Regulation of PLK1 Activity at G2/M Transition
REAC_Mitotic G2-G2/M phases
REAC_G2/M Transition
REAC_Cell Cycle Checkpoints
REAC_RHO GTPases Activate Formins
REAC_Mitotic Prometaphase
REAC_M Phase
GO:BP_nuclear division
GO:BP_mitotic cell cycle
REAC_Trafficking of GluR2-containing AMPA receptors
REAC_Signaling by NTRK3 (TRKC)
KEGG_ErbB signaling pathway
GO:BP_synaptic transmission, glutamatergic
GO:MF_AMPA glutamate receptor activity
GO:BP_chemical synaptic transmission
GO:BP_generation of neurons
GO:BP_neurogenesis
GO:MF_myosin binding
REAC_Clathrin-mediated endocytosis
GO:MF_guanyl-nucleotide exchange factor activity
GO:BP_neuron projection development
GO:BP_cell projection morphogenesis
GO:MF_actin binding
GO:MF_cytoskeletal protein binding
GO:BP_nervous system development
GO:BP_glucose metabolic process
GO:BP_positive regulation of cell adhesion
REAC_Signaling by Interleukins
GO:BP_blood vessel morphogenesis
KEGG_Mineral absorption
GO:BP_carbohydrate biosynthetic process
KEGG_HIF-1 signaling pathway
GO:BP_cellular response to cytokine stimulus
GO:MF_sodium ion transmembrane transporter activity
GO:MF_transforming growth factor beta-activated receptor activity
GO:BP_vascular process in circulatory system
GO:MF_adenylate cyclase regulator activity
GO:MF_metal ion transmembrane transporter activity
negative_log10_of_adjusted_p_value
D
Geneset Enrichment analysis
Color Key and Histogram
Column Z-Score
V_Mesenchymal
W_Mesenchymal.mGSC.
N_MES1
Gill_ReactiveAstrocytes
N_MES2
N_AC
V_classical
Gobin_G1
N_G1.S
N_G2.M
Gobin_G3
W_Proneuronal.pGSC.
SIMS_GBM
N_OPC
N_NPC1
Gill_OPC
N_NPC2
E
P_gl_PN2
P_gl_PN1
R_gl_PN2
R_gl_PN1
P_gl_Pro2
R_gl_Pro2
P_gl_Pro1
R_gl_Pro1
P_gl_Mes2
R_gl_Mes2
P_gl_Mes1
R_gl_Mes1
Primary
Recurrent
F
Percent Expressed
Average Expression
TOP2A SMC4 CENPF AURKB ATAD2 FANCA XRCC2 HELLS LIFR SLC1A3 CTNND2 MSI2 FTL VIM SAT1 CLU DOCK10 CTNNA3 PLP1 PLCL1 NRXN1 MEG3 SOX4 THSD7A

Supplementary Figure 3: snRNAseq-derived transcriptional states of putative neoplastic nuclei from post-treatment recurrent IDH-wildtype GBM samples

a) Uniform-manifold approximation and projection (UMAP) graphs showing putative neoplastic (CNVpos) and non-neoplastic (CNVneg) nuclei from the eight post-treatment recurrent IDH-wildtype glioblastoma cases. **a**) UMAP plot showing all putative CNVpos nuclei from the eight recurrent glioma cases aligned and projected in shared UMAP spaces. The nuclei are color-coded by glioma state: Oligodendrocyte-progenitor-like (proneural - gl_PN1), Neural-progenitor-like (proneural - gl_PN2), Mesenchymal/astrocyte like (gl_Mes1 and gl_Mes2), and proliferative (gl_Pro1 & gl_Pro2). **c**) Gene ontology (GO) term enrichment analysis (KEGG and REACTOME pathways and biological process GO) of the major terms enriched in glioma state top gene markers. The bars represent the negative log10 of the false discovery rate adjusted p.value and are color-coded as in B. **d**) Geneset enrichment analysis (GSEA) of selected genesets from Verhaak et al. 2009, Gobin M et al 2019, Gill et al 2014, and Neftel et al. 2019 showing enrichment of genes specific for states described in the literature in our described glioma states. **e**) Correlation heatmap between glioma states in primary and post-treatment recurrent GBM based on expression on glioma state marker genes. The size and color of the circles denote the strength of correlation. **f**) Gene expression dot plots showing select gene marker expression in glioma states.

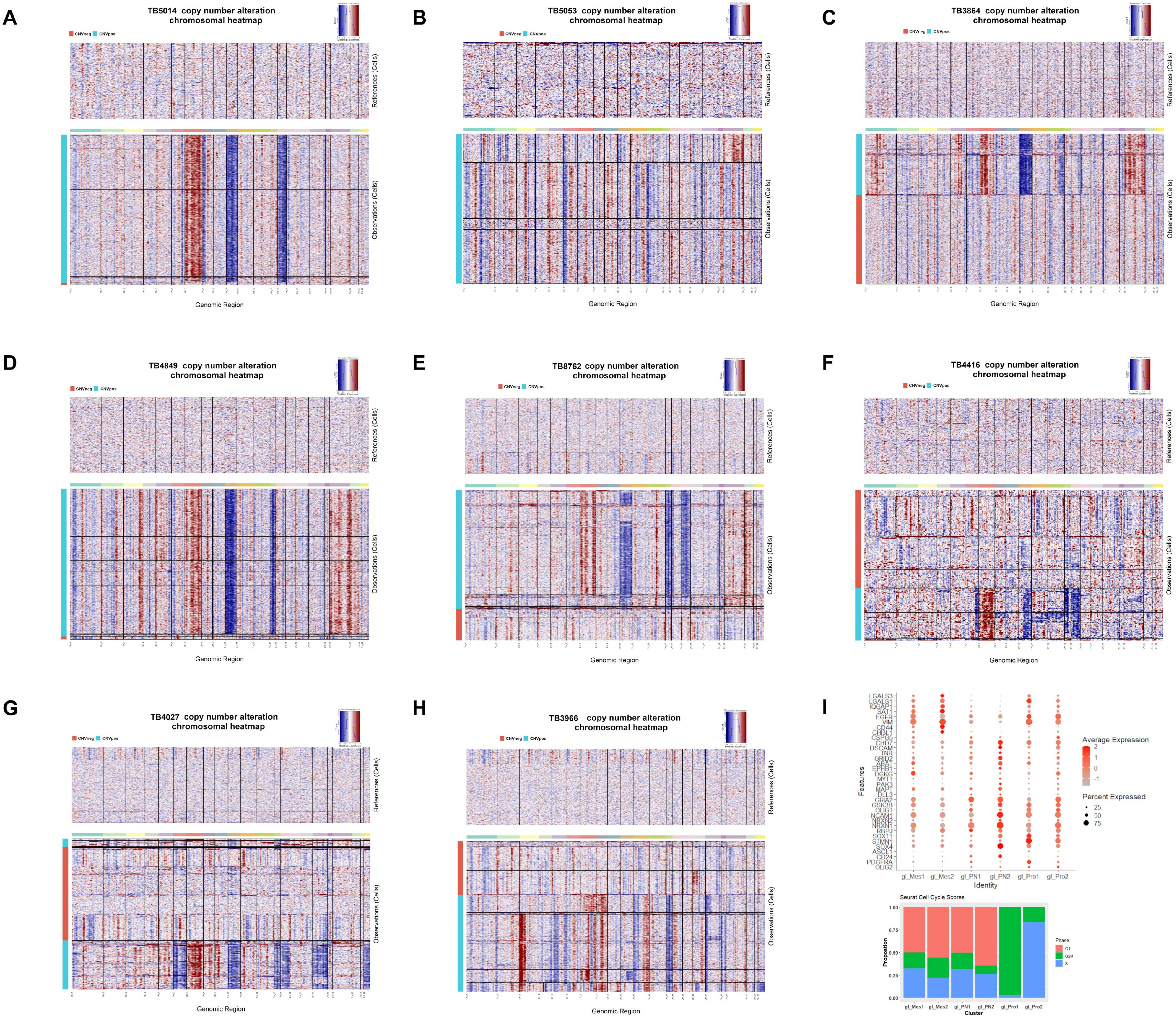

A
TB5014 copy number alteration chromosomal heatmap
B
TB5053 copy number alteration chromosomal heatmap
C
TB3864 copy number alteration chromosomal heatmap
D
TB4849 copy number alteration chromosomal heatmap
E
TB8762 copy number alteration chromosomal heatmap
F
TB4416 copy number alteration chromosomal heatmap
G
TB4027 copy number alteration chromosomal heatmap
H
TB3966 copy number alteration chromosomal heatmap
CNVneg
CNVpos
References (Cells)
Observations (Cells)
Genomic Region
I
Features
Identity
gl_Mes1
gl_Mes2
gl_PN1
gl_PN2
gl_Pro1
gl_Pro2
Average Expression
Percent Expressed
25
50
75
Seurat Cell Cycle Scores
Proportion
Cluster
Phase
G1
G2M
S

Supplementary Figure 4: CNV analysis of recurrent glioma samples

a-h) Large scale chromosomal copy number alterations were inferred from RNA expression using InferCNV R package. The heat maps show gains (red) and losses (blue) in case TB5014 (**a**), TB5053 **(b),** TB3864 (**c**), TB4898 (**d**), TB8762 **(e),** TB4416 (**f**), and TB4027 **(g),** and TB3966 (**h**). **i**) Dotplot showing expression of select set of markers of both primary and recurrent glioma states. The proportion of each glioma state in cell cycle phases as determined by Seurat cell-cycle scoring is shown on the bottom.

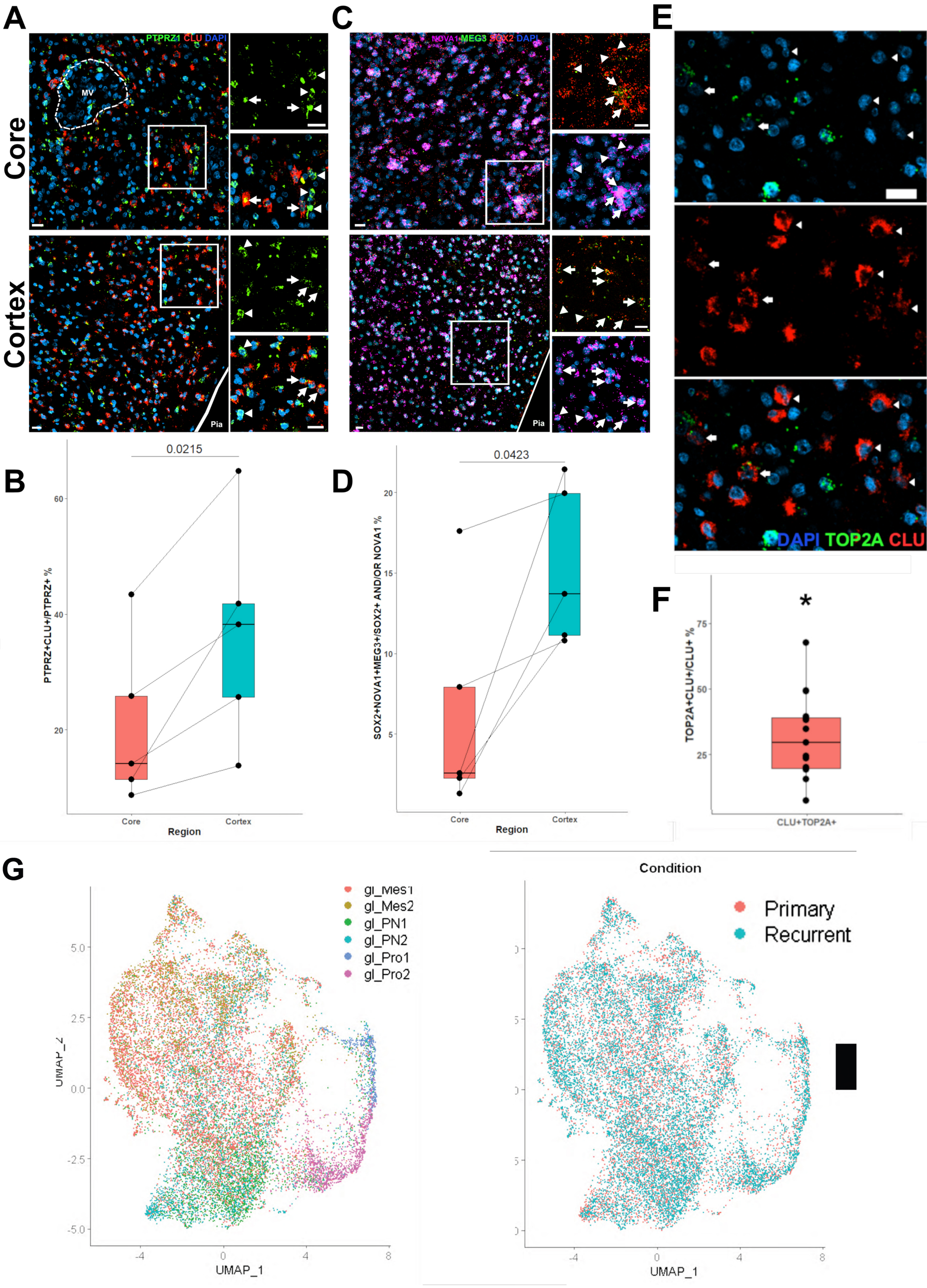

A
PTPRZ1 CLU DAPI
MV
Core
Cortex
Pia
C
NOVA1 MEG3 SOX2 DAPI
Pia
E
DAPI TOP2A CLU
B
0.0215
PTPRZ+CLU+/PTPRZ+ %
60
40
20
Core
Cortex
Region
D
0.0423
SOX2+NOVA1+MEG3+/SOX2+ AND/OR NOVA1 %
20
15
10
5
Core
Cortex
Region
F
*
TOP2A+CLU+/CLU+ %
75
50
25
CLU+TOP2A+
G
gl_Mes1
gl_Mes2
gl_PN1
gl_PN2
gl_Pro1
gl_Pro2
UMAP_2
5.0
2.5
0.0
-2.5
-5.0
-4
0
4
8
UMAP_1
Condition
Primary
Recurrent
UMAP_1

Supplementary Figure 5: The spatial landscape of glioma states across the cellular tumor and cortex

a) Confocal images showing optical sections of in situ hybridization for PTPRZ1 and CLU in the core (upper row) and cortex (lower row). The pial surface is outlined (lower row). High-power images of the insets show that PTPRZ1+ CLU+ cells (arrows) are more abundant in the cortex, while PTPRZ1+CLU- (arrowheads) are more numerous in the core. scale bars = 20 µm. M.V: Microvascular proliferation **b)** Quantification of PTPRZ1 and CLU expression across the core (orange boxplot) and cortex (green boxplot). The data is shown as boxplots with the 25th, 50th, and 75th percentiles indicated Two tailed paired t-test, n=5 independent samples external to the snRNAseq datasets. The p value is indicated. **c**) Confocal images showing optical sections of in situ hybridization for NOVA, SOX2, and MEG3 in the core (upper row) and cortex (lower row). The pial surface is outlined (lower row). High-power images of the insets show that NOVA1+SOX2+MEG3+ cells (arrows) are more abundant in the cortex, while MEG3- cells (arrowheads) are more numerous in the core. scale bars = 20 µm. **d**) Quantification of MEG3+NOVA1+SOX2+ cells as a proportion of all tumor cells (SOX2+ and/or NOVA1+) across the core (orange boxplot) and cortex (green boxplot). The data is shown as boxplots, with the 25th, 50th, and 75th percentiles indicated. One tailed paired t-test, n=5 independent samples. The p value is indicated. **e)** Confocal images showing optical sections of in situ hybridization for TOP2A and CLU in the GBM infiltrated tissue. Arrows indicate CLU+TOP2A+ cells, and arrowheads indicate CLU+TOP2A- cells. scale bar = 20 µm. **f)** Quantification of TOP2A and CLU expression. The percentage of TOP2A+CLU+/CLU+ cells is shown as a boxplot. One-sample t-test, n=5 independent tumor samples, three regions per sample. *=p value < 0.001. **g)** Integration of primary and recurrent GBM CNVpos nuclei color-coded by glioma state and condition.

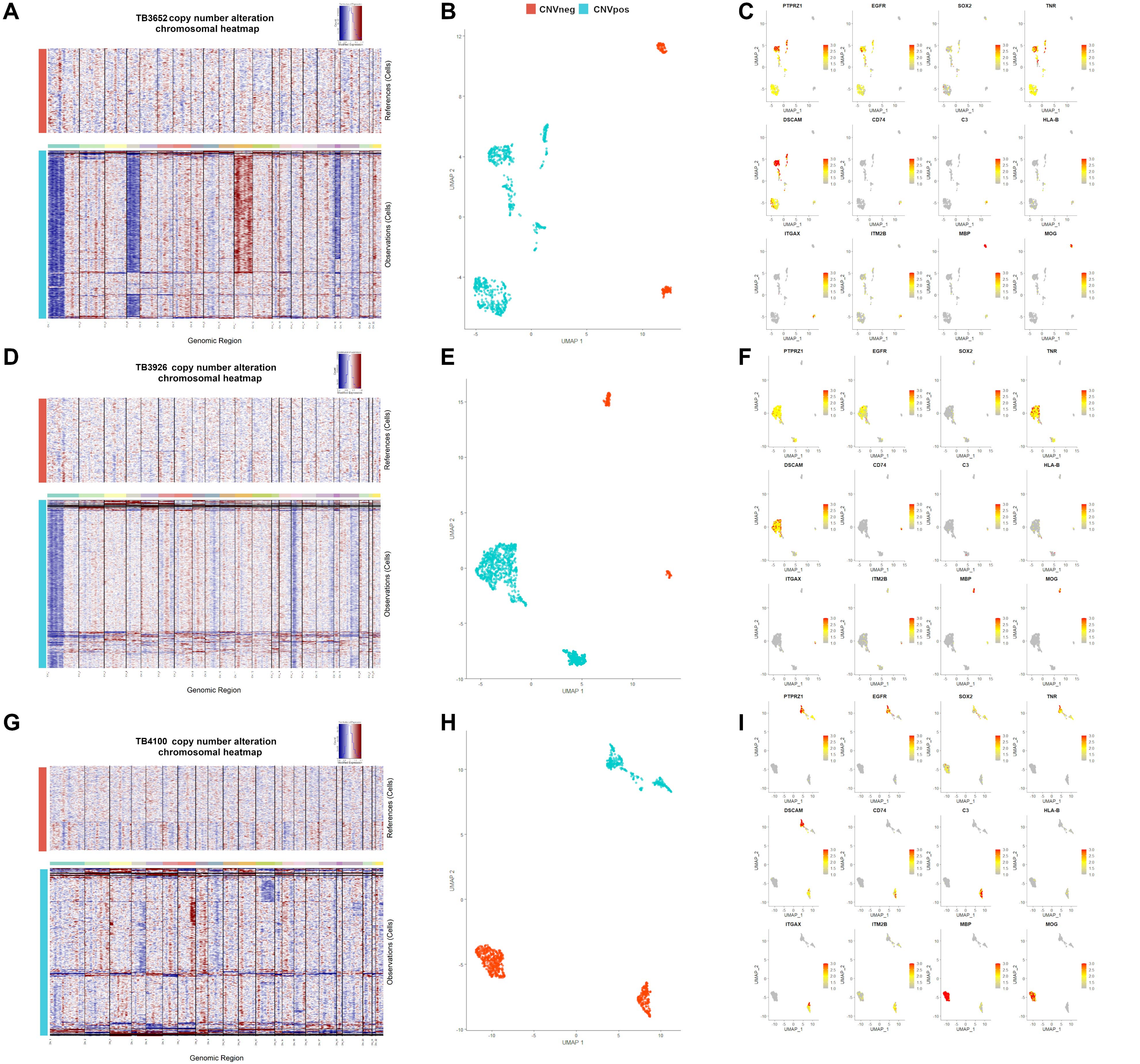
A
TB3652 copy number alteration chromosomal heatmap
References (Cells)
Observations (Cells)
Genomic Region
B
CNVneg
CNVpos
UMAP 1
UMAP 2
C
PTPRZ1
EGFR
SOX2
TNR
DSCAM
CD74
C3
HLA-B
ITGAX
ITM2B
MBP
MOG
D
TB3926 copy number alteration chromosomal heatmap
References (Cells)
Observations (Cells)
Genomic Region
E
UMAP 1
UMAP 2
F
G
TB4100 copy number alteration chromosomal heatmap
References (Cells)
Observations (Cells)
Genomic Region
H
UMAP 1
UMAP 2
I

Supplementary Figure 6: Analysis of Low-grade glioma samples using single nucleus RNAseq

Large scale chromosomal copy number alterations were inferred from RNA expression of cases TB3652 (**a**), TB3926 (**d**) – both IDH1-mutant oligodendrogliomas, and TB4100 (**g**) – IDH-mutant astrocytoma. Uniform manifold approximation and projection (UMAP) plots of the three cases are shown in panels **b**, **e**, and **h**, color-coded by copy number alteration status. Gene expression UMAPs showing markers of tumor cells (*PTPRZ1, EGFR, SOX2, TNR*, and *DSCAM*), immune cells (*CD74, C3, HLA-B, ITGAX, ITM2B*), and oligodendrocytes (*MBP, MOG*) of cases TB3652, TB3926, and TB4100 in panels **c, f,** and **i**, respectively.

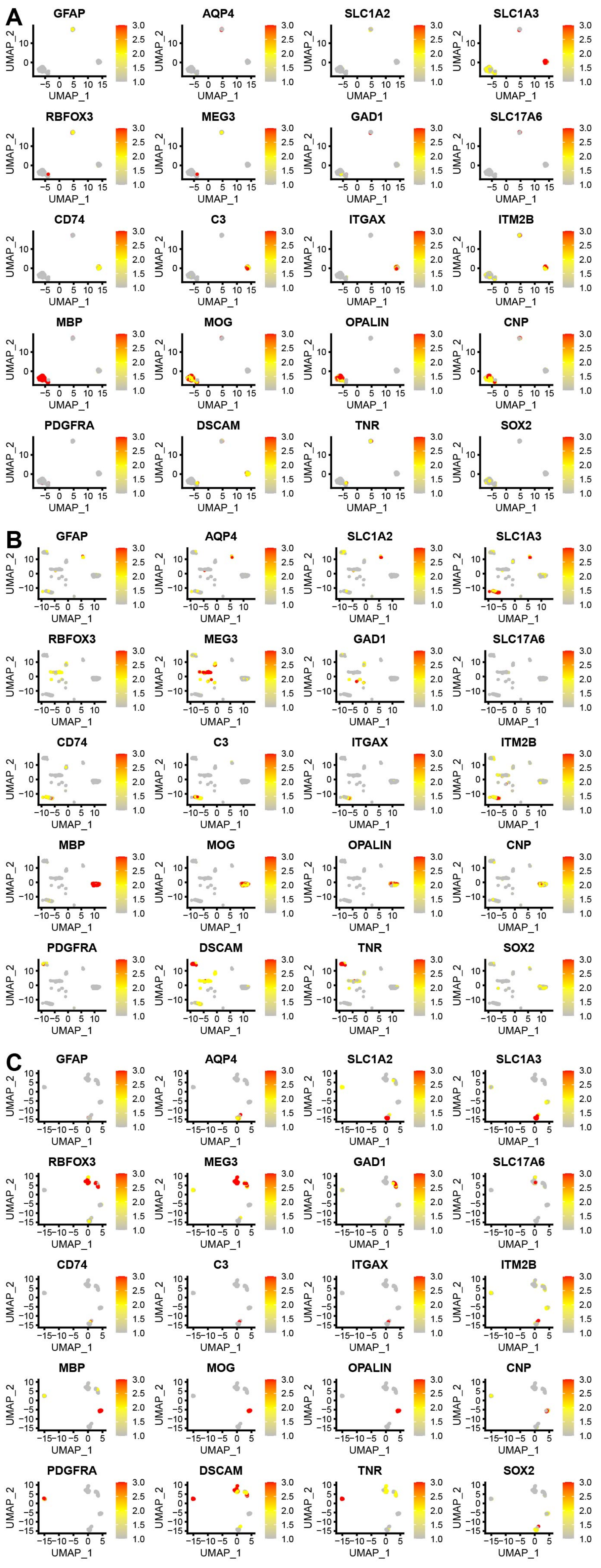
A
GFAP
AQP4
SLC1A2
SLC1A3
RBFOX3
MEG3
GAD1
SLC17A6
CD74
C3
ITGAX
ITM2B
MBP
MOG
OPALIN
CNP
PDGFRA
DSCAM
TNR
SOX2
B
GFAP
AQP4
SLC1A2
SLC1A3
RBFOX3
MEG3
GAD1
SLC17A6
CD74
C3
ITGAX
ITM2B
MBP
MOG
OPALIN
CNP
PDGFRA
DSCAM
TNR
SOX2
C
GFAP
AQP4
SLC1A2
SLC1A3
RBFOX3
MEG3
GAD1
SLC17A6
CD74
C3
ITGAX
ITM2B
MBP
MOG
OPALIN
CNP
PDGFRA
DSCAM
TNR
SOX2
UMAP_1
UMAP_2

Supplementary Figure 7: Analysis of Epilepsy samples using single nucleus RNAseq

a-c) Uniform-manifold approximation and projection (UMAP) graphs plots showing normalized gene expression of select lineage markers for cases TB4437 (**a**), TB4189 **(b),** TB4957 (**c**). The markers include astrocyte markers (*GFAP, AQP4, SLC1A2,* and *SLC1A3*), neuron makers (*RBFOX3, MEG3, GAD1, SLC17A6*), myeloid markers (*CD74, C3, ITGAX, ITM2B*), oligodendrocyte markers (*MBP, MOG, OPALIN, CNP*), and OPC markers (*PDGFRA, DSCAM, TNR,* and *SOX2*).

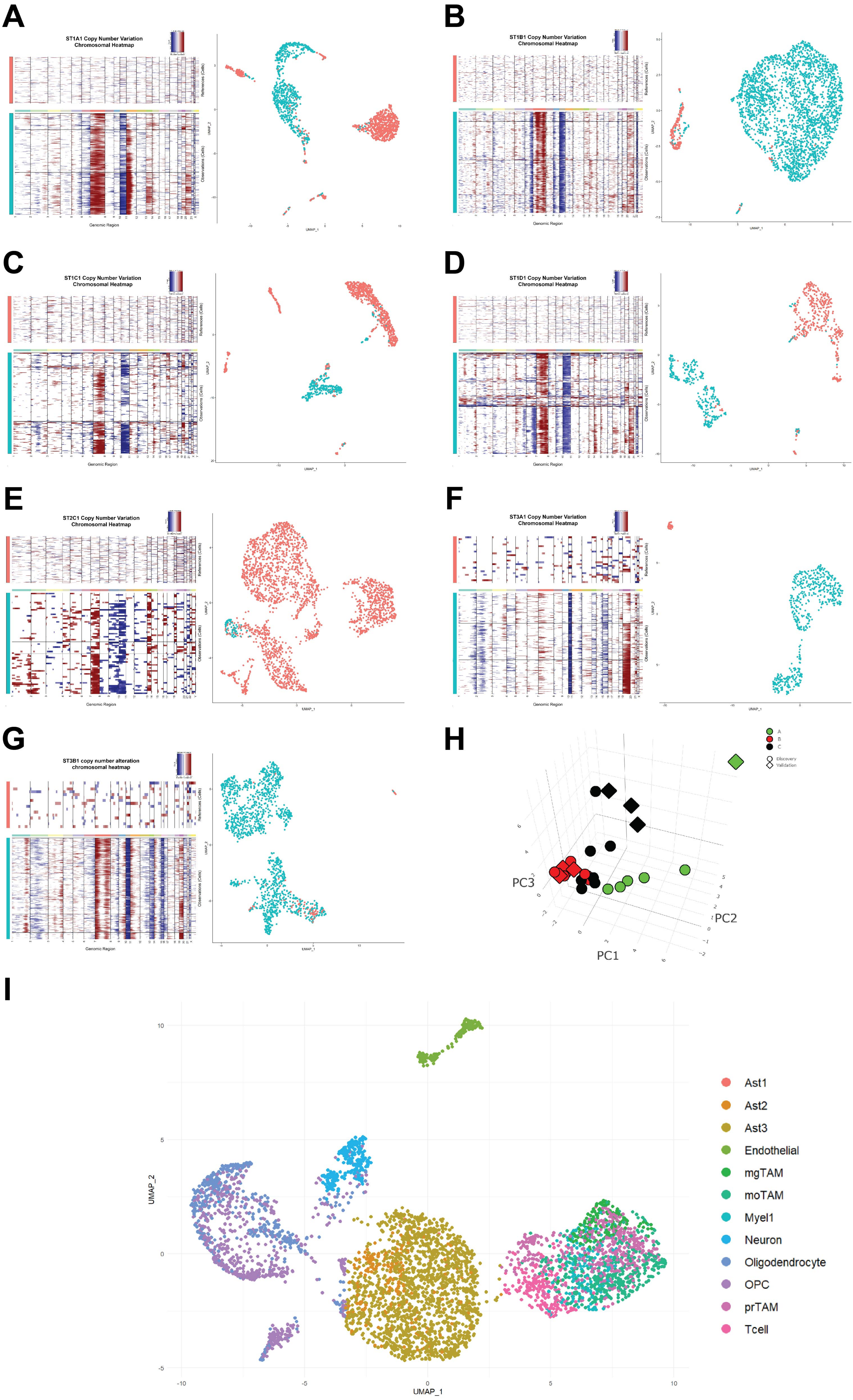
A
ST1A1 Copy Number Variation Chromosomal Heatmap
B
ST1B1 Copy Number Variation Chromosomal Heatmap
C
ST1C1 Copy Number Variation Chromosomal Heatmap
D
ST1D1 Copy Number Variation Chromosomal Heatmap
E
ST2C1 Copy Number Variation Chromosomal Heatmap
F
ST3A1 Copy Number Variation Chromosomal Heatmap
G
ST3B1 copy number alteration chromosomal heatmap
References (Cells)
Observations (Cells)
Genomic Region
UMAP_1
UMAP_2
H
A
B
C
Discovery
Validation
PC1
PC2
PC3
I
Ast1
Ast2
Ast3
Endothelial
mgTAM
moTAM
Myel1
Neuron
Oligodendrocyte
OPC
prTAM
Tcell

Supplementary Figure 8: Analysis of Validation single nuclei data set

a-g) Heatmaps and UMAP projections of single nuclei extracted from separate sections of tissue that underwent spatial transcriptomics analysis showing copy number variation analysis using the InferCNV R package. Nuclei colored red were classified CNVneg and nuclei colored blue were classified CNVpos. CNVpos and CNVneg cells across samples were integrated and clustered separately before being categorized into specific cell types (see methods.) **h)** The composition of each validation dataset sample was determined and each validation sample was projected onto the PCA axes used to classify the discovery dataset samples. **i)** UMAP projection of all CNVneg nuclei across the validation data set, colored by cell type identity.

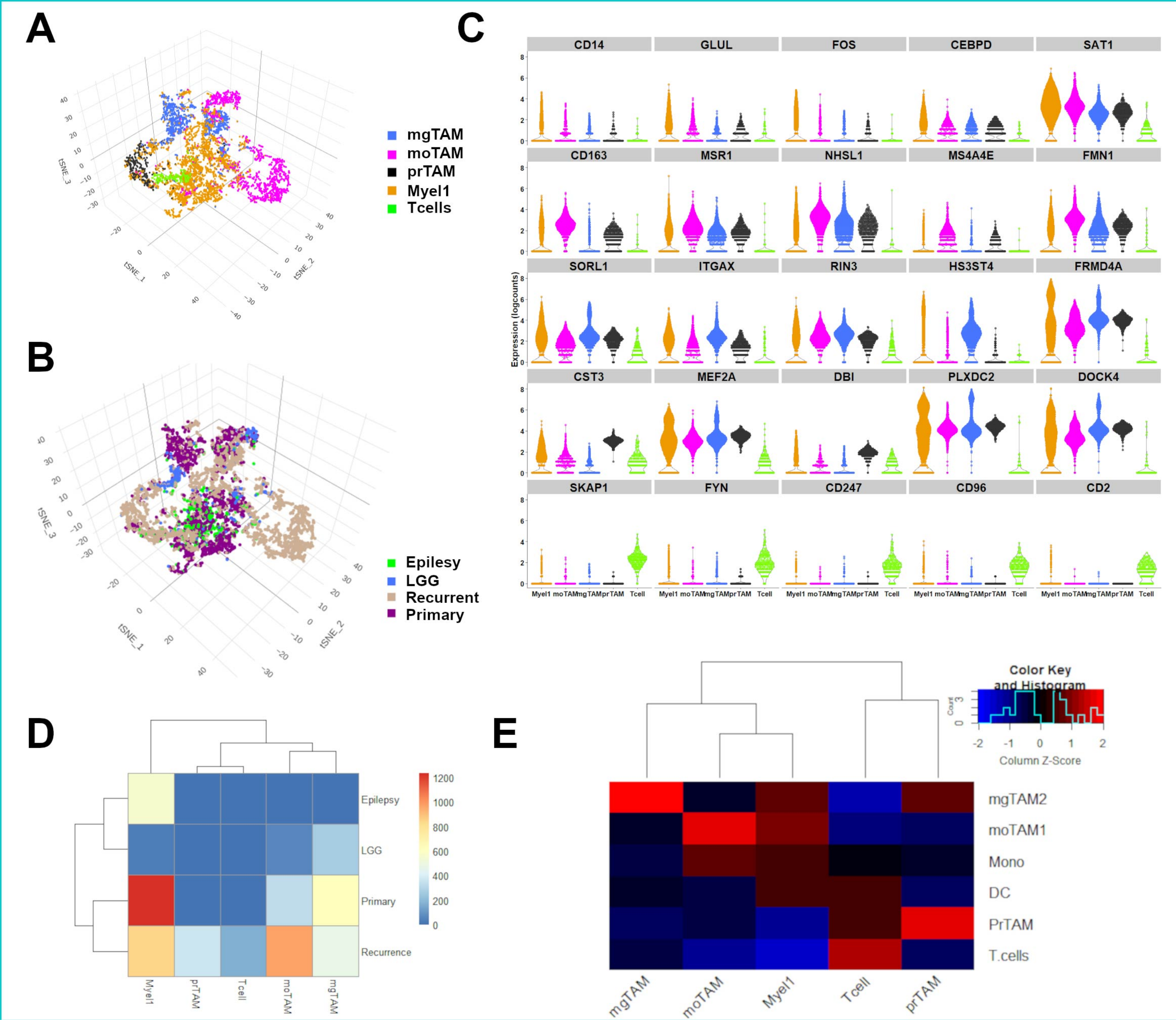
A
B
C
D
E
mgTAM
moTAM
prTAM
Myel1
Tcells
tSNE_1
tSNE_2
tSNE_3
Epilesy
LGG
Recurrent
Primary
CD14
GLUL
FOS
CEBPD
SAT1
CD163
MSR1
NHSL1
MS4A4E
FMN1
SORL1
ITGAX
RIN3
HS3ST4
FRMD4A
CST3
MEF2A
DBI
PLXDC2
DOCK4
SKAP1
FYN
CD247
CD96
CD2
Expression (logcounts)
Myel1 moTAM mgTAM prTAM Tcell
Epilepsy
LGG
Primary
Recurrence
Myel1
prTAM
Tcell
moTAM
mgTAM
Color Key and Histogram
Count
Column Z-Score
mgTAM2
moTAM1
Mono
DC
PrTAM
T.cells
mgTAM
moTAM
Myel1
Tcell
prTAM

Supplementary Figure 9: *The transcriptional landscape of microglia in glioma*

a) Uniform-manifold approximation and projection (UMAP) graphs plots showing all myeloid nuclei from color-coded by cluster **(b)** and condition (primary glioma, recurrent glioma, low grade glioma (LGG), and epilepsy (**c**). Gene expression violin plots showing select gene marker expression for the immune cell clusters from top to bottom; Myel1, mgTAM, moTAM, prTAM, and T cells. **d**) Heatmap showing the proportion of nuclei in each cluster (columns) contributed by condition (rows). **e**) Heatmap showing the scaled enrichment scores of gene sets derived from Movahedi et al 2021 in the nuclei pooled from each myeloid cluster.

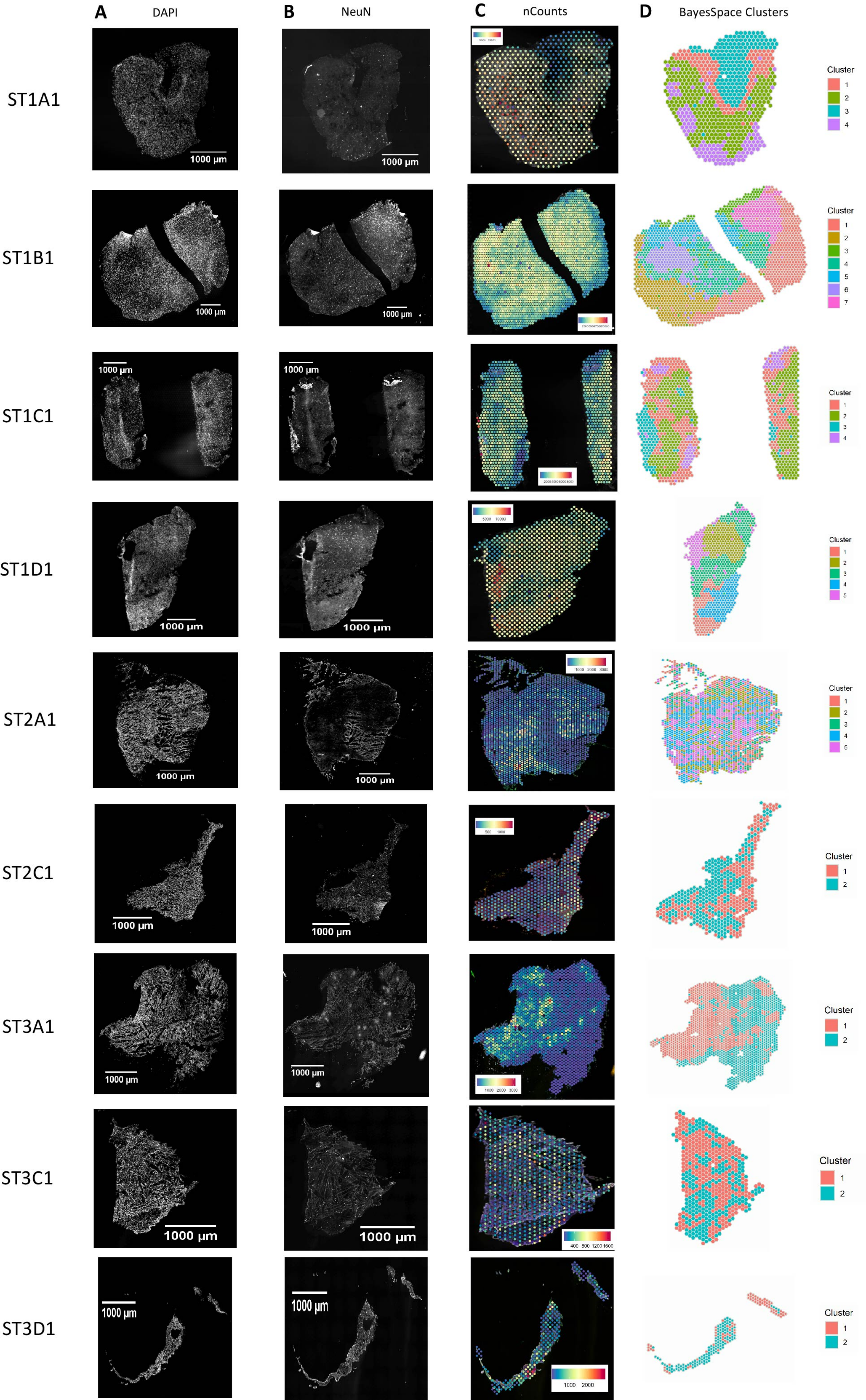

A
DAPI
B
NeuN
C
nCounts
D
BayesSpace Clusters
ST1A1
ST1B1
ST1C1
ST1D1
ST2A1
ST2C1
ST3A1
ST3C1
ST3D1
1000 µm
Cluster

Supplementary Figure 10: Spatial transcriptomics samples.

a-b) DAPI and NeuN staining of ST samples (n= 9 samples; scale bar equals 1000µm). **c)** Spatial transcriptomic images annotated with number of unique genes observed at each spot (n= 9 samples). **d)** BayesSpace-generated clusters overlaid on each ST sample. The number of clusters for each sample was determined through maximization of the modified Bayesian Information Criterion (MBIC).

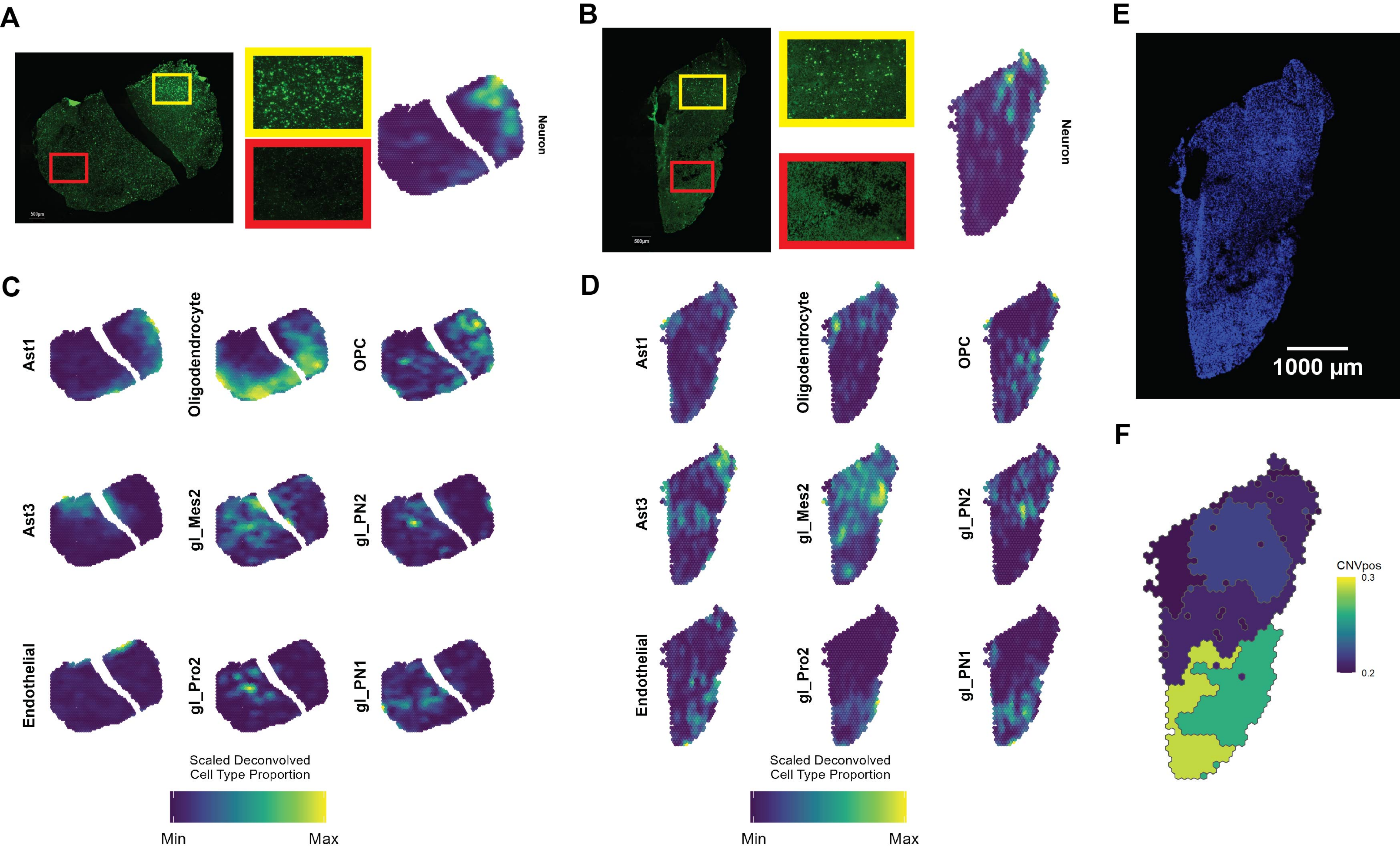

A
500µm
Neuron
B
500µm
Neuron
E
1000 µm
C
Ast1
Oligodendrocyte
OPC
Ast3
gl_Mes2
gl_PN2
Endothelial
gl_Pro2
gl_PN1
Scaled Deconvolved
Cell Type Proportion
Min
Max
D
Ast1
Oligodendrocyte
OPC
Ast3
gl_Mes2
gl_PN2
Endothelial
gl_Pro2
gl_PN1
Scaled Deconvolved
Cell Type Proportion
Min
Max
F
CNVpos
0.3
0.2

Supplementary Figure 11: Deconvolution of Spatial Transcriptomic Samples.

a-b) Representative images of staining for NeuN alongside the deconvolved proportions of Neurons in samples ST1B1 and ST1D1 (scale bar equals 500µm.) Red and yellow insets show detailed view of NeuN staining that correlates with the patterns of heterogeneity shown by the deconvolved proportion of neurons. **c-d)** Deconvolved proportions of selected cell types that comprise each tissue state projected onto maps of samples ST1B1 and ST1D1 respectively. **e)** Sample ST1D1 stained for DAPI (scale bar equals 1000µm) and **f**) sample ST1D1 segmented by BayesSpace into clusters and shaded based on the proportion of CNVpos cell types determined by deconvolution. Clusters with a higher density of nuclei were correlated with a higher proportion of CNVpos cell types across the dataset.

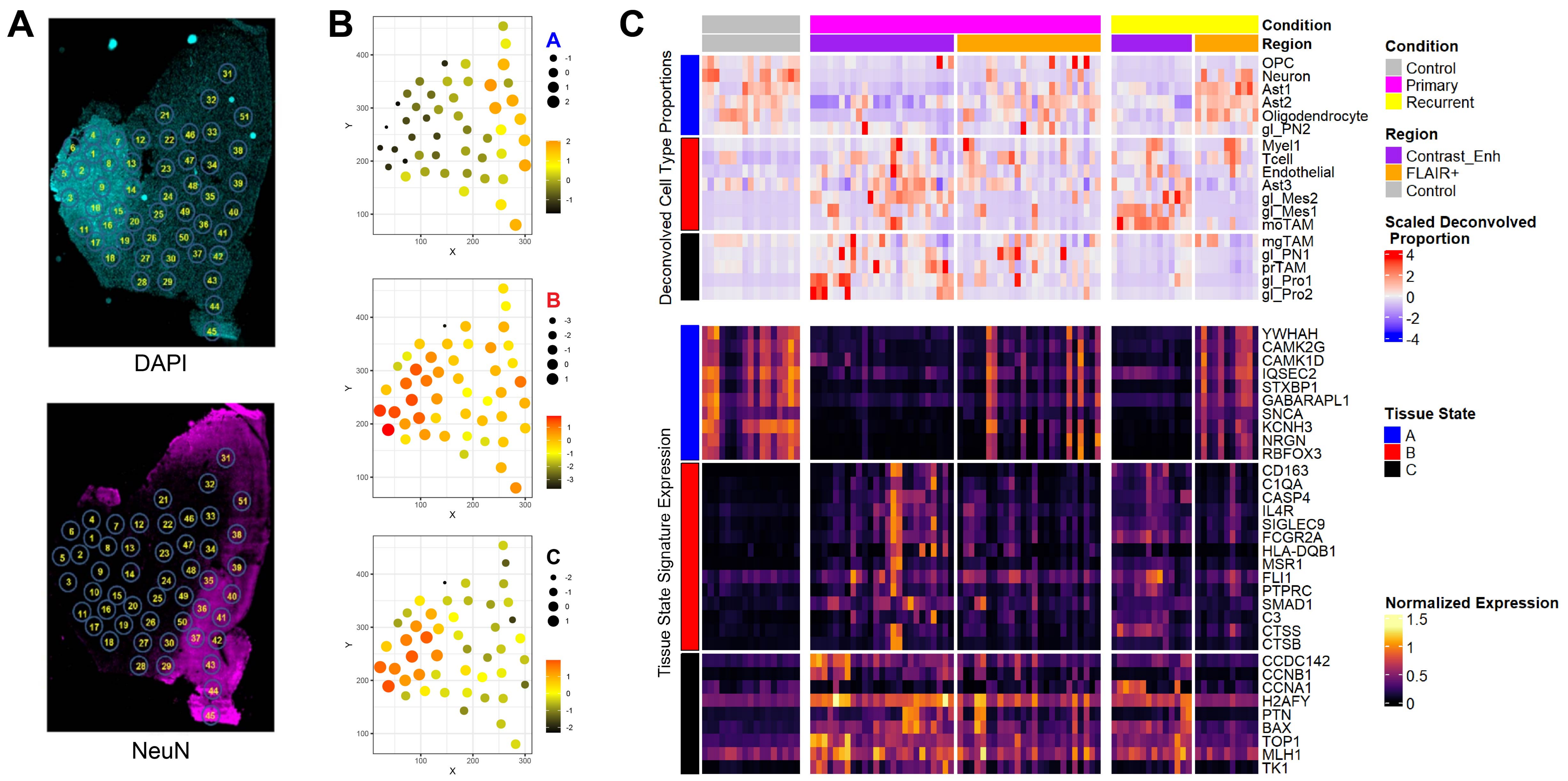
A
DAPI
NeuN
B
A
B
C
X
Y
C
Condition
Region
Deconvolved Cell Type Proportions
OPC
Neuron
Ast1
Ast2
Oligodendrocyte
gl_PN2
Myel1
Tcell
Endothelial
Ast3
gl_Mes2
gl_Mes1
moTAM
mgTAM
gl_PN1
prTAM
gl_Pro1
gl_Pro2
Tissue State Signature Expression
YWHAH
CAMK2G
CAMK1D
IQSEC2
STXBP1
GABARAPL1
SNCA
KCNH3
NRGN
RBFOX3
CD163
C1QA
CASP4
IL4R
SIGLEC9
FCGR2A
HLA-DQB1
MSR1
FLI1
PTPRC
SMAD1
C3
CTSS
CTSB
CCDC142
CCNB1
CCNA1
H2AFY
PTN
BAX
TOP1
MLH1
TK1
Condition
Control
Primary
Recurrent
Region
Contrast_Enh
FLAIR+
Control
Scaled Deconvolved Proportion
Tissue State
A
B
C
Normalized Expression

Supplementary Figure 12: The spatial landscape of glioma margins.

a) Outline of spatial transcriptomic analysis of infiltrating GBM. DAPI (left) and NeuN (right) immunostains of frozen sections from case PO2, for which snRNAseq was done. Each circle represents a biopsy on which bulk RNAseq was done. After the biopsies were taken, the specimen was bisected along the dashed white line (y-axis) and subjected to snRNASeq. **b**) Enrichment analysis of each of the spatially mapped biopsies using the genesets of the three compositional clusters (see text for details) displaying normalized single sample GSEA enrichment scores for the tumor cluster (C - upper panel), the tumor-reactive cluster (B– middle panel), and the normal brain cluster (A – lower panel). The enrichment scores are coded by color and size. The normalized RNA data for the spatial biopsy map is available in an interactive web interface at https://vmenon.shinyapps.io/gbm_expression/. **c**) Heatmaps showing the scaled proportion of 18 cell types obtained by deconvolution and corresponding normalized expression of markers from tissue-state signatures for each sample as applied to the Gill et al. 2014 MRI localized biopsy dataset (n=92). Results are stratified by condition and by MRI localization. Cell types/markers are annotated with their corresponding tissue-states on the left.

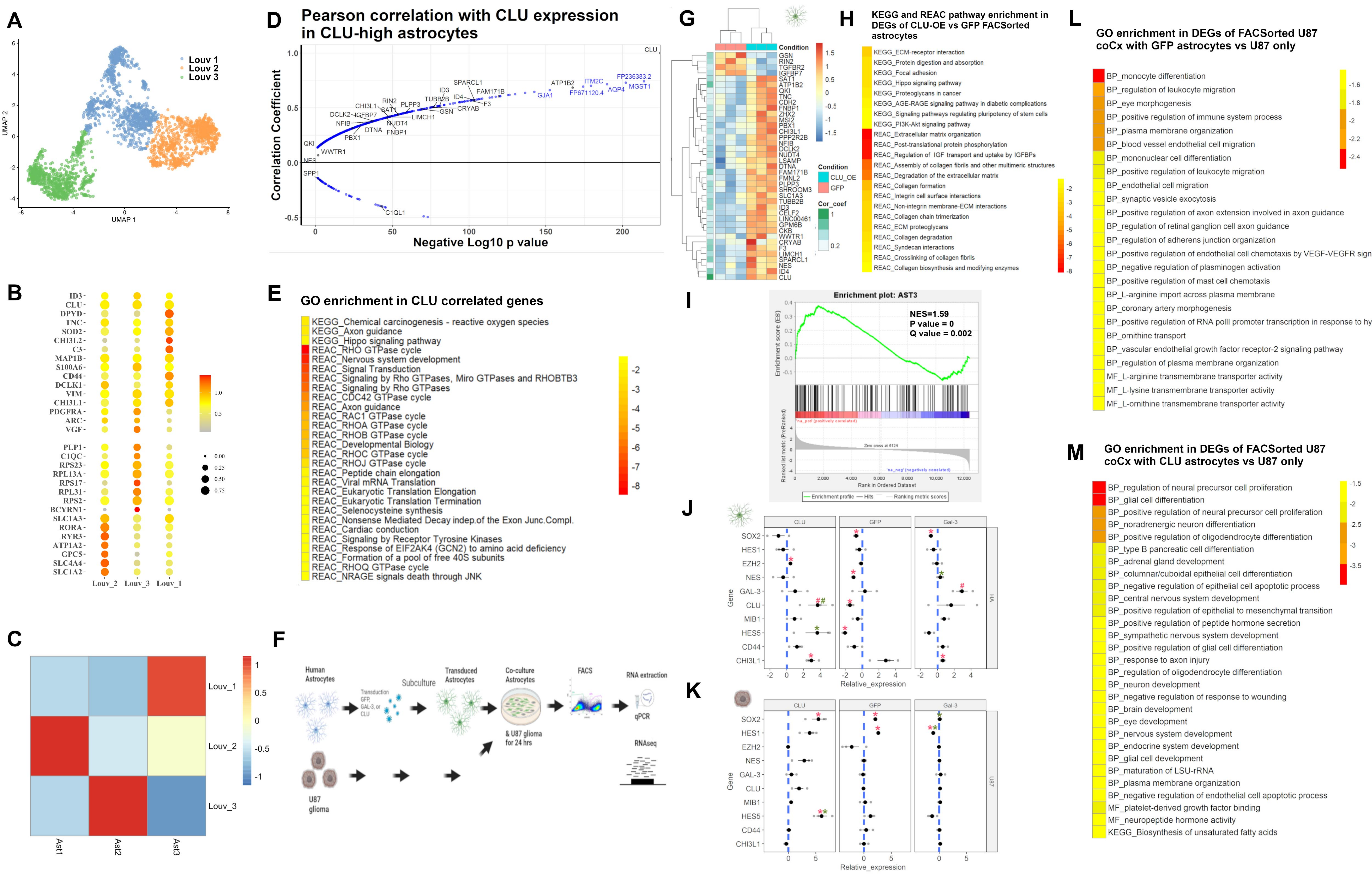
A
Louv 1
Louv 2
Louv 3
UMAP 1
UMAP 2
B
ID3
CLU
DPYD
TNC
SOD2
CHI3L2
C3
MAP1B
S100A6
CD44
DCLK1
VIM
CHI3L1
PDGFRA
ARC
VGF
PLP1
C1QC
RPS23
RPL13A
RPS17
RPL31
RPS2
BCYRN1
SLC1A3
RORA
RYR3
ATP1A2
GPC5
SLC4A4
SLC1A2
Louv_2
Louv_3
Louv_1
C
Louv_1
Louv_2
Louv_3
Ast1
Ast2
Ast3
D
Pearson correlation with CLU expression in CLU-high astrocytes
Correlation Coefficient
Negative Log10 p value
E
GO enrichment in CLU correlated genes
KEGG_Chemical carcinogenesis - reactive oxygen species
KEGG_Axon guidance
KEGG_Hippo signaling pathway
REAC_RHO GTPase cycle
REAC_Nervous system development
REAC_Signal Transduction
REAC_Signaling by Rho GTPases, Miro GTPases and RHOBTB3
REAC_Signaling by Rho GTPases
REAC_CDC42 GTPase cycle
REAC_Axon guidance
REAC_RAC1 GTPase cycle
REAC_RHOA GTPase cycle
REAC_RHOB GTPase cycle
REAC_Developmental Biology
REAC_RHOC GTPase cycle
REAC_RHOJ GTPase cycle
REAC_Peptide chain elongation
REAC_Viral mRNA Translation
REAC_Eukaryotic Translation Elongation
REAC_Eukaryotic Translation Termination
REAC_Selenocysteine synthesis
REAC_Nonsense Mediated Decay indep.of the Exon Junc.Compl.
REAC_Cardiac conduction
REAC_Signaling by Receptor Tyrosine Kinases
REAC_Response of EIF2AK4 (GCN2) to amino acid deficiency
REAC_Formation of a pool of free 40S subunits
REAC_RHOQ GTPase cycle
REAC_NRAGE signals death through JNK
F
Human Astrocytes
Transduction GFP, GAL-3, or CLU
Subculture
Transduced Astrocytes
Co-culture Astrocytes
& U87 glioma for 24 hrs
FACS
RNA extraction
qPCR
RNAseq
U87 glioma
G
Condition
CLU_OE
GFP
Cor_coef
H
KEGG and REAC pathway enrichment in DEGs of CLU-OE vs GFP FACSorted astrocytes
KEGG_ECM-receptor interaction
KEGG_Protein digestion and absorption
KEGG_Focal adhesion
KEGG_Hippo signaling pathway
KEGG_Proteoglycans in cancer
KEGG_AGE-RAGE signaling pathway in diabetic complications
KEGG_Signaling pathways regulating pluripotency of stem cells
KEGG_PI3K-Akt signaling pathway
REAC_Extracellular matrix organization
REAC_Post-translational protein phosphorylation
REAC_Regulation of IGF transport and uptake by IGFBPs
REAC_Assembly of collagen fibrils and other multimeric structures
REAC_Degradation of the extracellular matrix
REAC_Collagen formation
REAC_Integrin cell surface interactions
REAC_Non-integrin membrane-ECM interactions
REAC_Collagen chain trimerization
REAC_ECM proteoglycans
REAC_Collagen degradation
REAC_Syndecan interactions
REAC_Crosslinking of collagen fibrils
REAC_Collagen biosynthesis and modifying enzymes
I
Enrichment plot: AST3
NES=1.59
P value = 0
Q value = 0.002
Rank in Ordered Dataset
Enrichment profile
Hits
Ranking metric scores
J
CLU
GFP
Gal-3
HA
Gene
Relative_expression
K
U87
L
GO enrichment in DEGs of FACSorted U87 coCx with GFP astrocytes vs U87 only
BP_monocyte differentiation
BP_regulation of leukocyte migration
BP_eye morphogenesis
BP_positive regulation of immune system process
BP_plasma membrane organization
BP_blood vessel endothelial cell migration
BP_mononuclear cell differentiation
BP_positive regulation of leukocyte migration
BP_endothelial cell migration
BP_synaptic vesicle exocytosis
BP_positive regulation of axon extension involved in axon guidance
BP_regulation of retinal ganglion cell axon guidance
BP_regulation of adherens junction organization
BP_positive regulation of endothelial cell chemotaxis by VEGF-VEGFR sign
BP_negative regulation of plasminogen activation
BP_positive regulation of mast cell chemotaxis
BP_L-arginine import across plasma membrane
BP_coronary artery morphogenesis
BP_positive regulation of RNA polII promoter transcription in response to hy
BP_ornithine transport
BP_vascular endothelial growth factor receptor-2 signaling pathway
BP_regulation of plasma membrane organization
MF_L-arginine transmembrane transporter activity
MF_L-lysine transmembrane transporter activity
MF_L-ornithine transmembrane transporter activity
M
GO enrichment in DEGs of FACSorted U87 coCx with CLU astrocytes vs U87 only
BP_regulation of neural precursor cell proliferation
BP_glial cell differentiation
BP_positive regulation of neural precursor cell proliferation
BP_noradrenergic neuron differentiation
BP_positive regulation of oligodendrocyte differentiation
BP_type B pancreatic cell differentiation
BP_adrenal gland development
BP_columnar/cuboidal epithelial cell differentiation
BP_negative regulation of epithelial cell apoptotic process
BP_central nervous system development
BP_positive regulation of epithelial to mesenchymal transition
BP_positive regulation of peptide hormone secretion
BP_sympathetic nervous system development
BP_positive regulation of glial cell differentiation
BP_response to axon injury
BP_regulation of oligodendrocyte differentiation
BP_neuron development
BP_negative regulation of response to wounding
BP_brain development
BP_eye development
BP_nervous system development
BP_endocrine system development
BP_glial cell development
BP_maturation of LSU-rRNA
BP_plasma membrane organization
BP_negative regulation of endothelial cell apoptotic process
MF_platelet-derived growth factor binding
MF_neuropeptide hormone activity
KEGG_Biosynthesis of unsaturated fatty acids

Supplementary Figure 13: Astrocytes influence glioma gene expression.

a) UMAP projection of astrocytes clustered using Louvain clustering on shared-nearest neighbor graphs created using igraph – k=500. Three clusters are shown. **b)** Dotplots of select cluster markers. **c**) Scaled overlap between astrocyte identities designated using geneset enrichment clustering (described in figure 2) versus unbiased clustering described in **a** - scaled by column. **d**) Correlation analysis showing the Pearson correlation coefficients (y-axis) of genes that correlate with *CLU* expression in *CLU*-high astrocytes – defined as having normalized expression in the third or fourth quantiles. The p value (against the null hypothesis of no correlation) is shown on the x-axis. **e**) Gene ontology enrichment analysis of genes that positively correlate with *CLU* from A. The negative log10 p value of enrichment is indicated. **f**) Outline of astrocyte glioma co-culture experiment. This panel was created with BioRender.com. **g**) Heatmap of normalized expression of astrocytes with *CLU* over-expression (*CLU*_OE) versus GFP control astrocytes co-cultured with U87 glioma cells. The genes are annotated by the correlation coefficient with *CLU* from A. **h**) KEGG/Reactome pathway enrichment in genes differentially expressed in FACSorted *CLU*_OE astrocytes versus *GFP* astrocytes after co-culture with U87 glioma. The negative log10 p value of enrichment is indicated. **i)** Pre-ranked GSEA in differentially expressed genes between *CLU*_OE and *GFP* control astrocytes for the Ast3 gene signature. Normalized Enrichment Score (NES) p-values (two tailed t-test) and FDR-adjusted q-values are displayed. **j-k**) Real-time quantitative PCR of select genes from *CLU*_OE, *GFP*, or *LGALS3*_OE astrocytes (**j**) and U87 glioma (**k**) FACSorted from co-culture. Genes were selected for relevance to Ast3 signature orto glioma biology). The y-axis shows log normalized delta-delta Ct values. The p values are indicated. #: one-tailed paired t-test, *: two-tailed paired t-test. n = 3 independent FACsorting experiments. Error bars indicate standard error of the mean. Comparison group: control astrocytes not co-cultured with glioma for "red" * or #; control astrocytes co-cultured with U87 cells for "green" * or #. **l-m**) Gene ontology enrichment of genes differentially expressed in U87 cells co-cultured with *GFP* astrocytes (versus U87 cells not co-cultured **l**) or co-cultured with *CLU*_OE astrocytes versus U87 glioma co-cultured with *GFP* astrocytes (**m**). The negative log10 p value of enrichment is indicated.

Supplementary References

1. Tickle, T., Tirosh, I., Georgescu, C., Brown, M., and Haas, B. (2019). inferCNV of the Trinity CTAT Project.
2. Levitin, H.M., Yuan, J., Cheng, Y.L., Ruiz, F.J., Bush, E.C., Bruce, J.N., Canoll, P., Iavarone, A., Lasorella, A., Blei, D.M., and Sims, P.A. (2019). De novo gene signature identification from single-cell RNA-seq with hierarchical Poisson factorization. Mol Syst Biol *15*, e8557. 10.15252/msb.20188557.
3. Yuan, J., Levitin, H.M., Frattini, V., Bush, E.C., Boyett, D.M., Samanamud, J., Ceccarelli, M., Dovas, A., Zanazzi, G., Canoll, P., et al. (2018). Single-cell transcriptome analysis of lineage diversity in high-grade glioma. Genome Med *10*, 57. 10.1186/s13073-018-0567-9.
4. Butler, A., Hoffman, P., Smibert, P., Papalexi, E., and Satija, R. (2018). Integrating single-cell transcriptomic data across different conditions, technologies, and species. Nat Biotechnol *36*, 411-420. 10.1038/nbt.4096.
5. Verhaak, R.G., Hoadley, K.A., Purdom, E., Wang, V., Qi, Y., Wilkerson, M.D., Miller, C.R., Ding, L., Golub, T., Mesirov, J.P., et al. (2010). Integrated genomic analysis identifies clinically relevant subtypes of glioblastoma characterized by abnormalities in PDGFRA, IDH1, EGFR, and NF1. Cancer cell *17*, 98-110. 10.1016/j.ccr.2009.12.020.
6. Neftel, C., Laffy, J., Filbin, M.G., Hara, T., Shore, M.E., Rahme, G.J., Richman, A.R., Silverbush, D., Shaw, M.L., Hebert, C.M., et al. (2019). An Integrative Model of Cellular States, Plasticity, and Genetics for Glioblastoma. Cell *178*, 835-849 e821. 10.1016/j.cell.2019.06.024.
7. Gill, B.J., Pisapia, D.J., Malone, H.R., Goldstein, H., Lei, L., Sonabend, A., Yun, J., Samanamud, J., Sims, J.S., Banu, M., et al. (2014). MRI-localized biopsies reveal subtype-specific differences in molecular and cellular composition at the margins of glioblastoma. Proceedings of the National Academy of Sciences of the United States of America *111*, 12550-12555. 10.1073/pnas.1405839111.
8. Gobin, M., Nazarov, P.V., Warta, R., Timmer, M., Reifenberger, G., Felsberg, J., Vallar, L., Chalmers, A.J., Herold-Mende, C.C., Goldbrunner, R., et al. (2019). A DNA Repair and Cell-Cycle Gene Expression Signature in Primary and Recurrent Glioblastoma: Prognostic Value and Clinical Implications. Cancer Res *79*, 1226-1238. 10.1158/0008-5472.CAN-18-2076.
9. Wang, L., Babikir, H., Muller, S., Yagnik, G., Shamardani, K., Catalan, F., Kohanbash, G., Alvarado, B., Di Lullo, E., Kriegstein, A., et al. (2019). The Phenotypes of Proliferating Glioblastoma Cells Reside on a Single Axis of Variation. Cancer Discov *9*, 1708-1719. 10.1158/2159-8290.CD-19-0329.
10. Wang, Q., Hu, B., Hu, X., Kim, H., Squatrito, M., Scarpace, L., deCarvalho, A.C., Lyu, S., Li, P., Li, Y., et al. (2017). Tumor Evolution of Glioma-Intrinsic Gene Expression Subtypes Associates with Immunological Changes in the Microenvironment. Cancer cell *32*, 42-56 e46. 10.1016/j.ccell.2017.06.003.
11. Lopez, K.A., Tannenbaum, A.M., Assanah, M.C., Linskey, K., Yun, J., Kangarlu, A., Gil, O.D., Canoll, P., and Bruce, J.N. (2011). Convection-enhanced delivery of topotecan into a PDGF-driven model of glioblastoma prolongs survival and ablates both tumor-initiating cells and recruited glial progenitors. Cancer Res *71*, 3963-3971. 10.1158/0008-5472.CAN-10-0906.
12. Markovic, D.S., Glass, R., Synowitz, M., Rooijen, N., and Kettenmann, H. (2005). Microglia stimulate the invasiveness of glioma cells by increasing the activity of metalloprotease-2. J Neuropathol Exp Neurol *64*, 754-762. 10.1097/01.jnen.0000178445.33972.a9.
13. Pombo Antunes, A.R., Scheyltjens, I., Lodi, F., Messiaen, J., Antoranz, A., Duerinck, J., Kancheva, D., Martens, L., De Vlaminck, K., Van Hove, H., et al. (2021). Single-cell profiling of myeloid cells in glioblastoma across species and disease stage reveals macrophage competition and specialization. Nature neuroscience *24*, 595-610. 10.1038/s41593-020-00789-y.

14. Aran, D., Looney, A.P., Liu, L., Wu, E., Fong, V., Hsu, A., Chak, S., Naikawadi, R.P., Wolters, P.J., Abate, A.R., et al. (2019). Reference-based analysis of lung single-cell sequencing reveals a transitional profibrotic macrophage. Nat Immunol *20*, 163-172. 10.1038/s41590-018-0276-y.
15. Bush, E.C., Ray, F., Alvarez, M.J., Realubit, R., Li, H., Karan, C., Califano, A., and Sims, P.A. (2017). PLATE-Seq for genome-wide regulatory network analysis of high-throughput screens. Nature communications *8*, 105. 10.1038/s41467-017-00136-z.
16. Chen, F., Swartzlander, D.B., Ghosh, A., Fryer, J.D., Wang, B., and Zheng, H. (2021). Clusterin secreted from astrocyte promotes excitatory synaptic transmission and ameliorates Alzheimer's disease neuropathology. Molecular neurodegeneration *16*, 5. 10.1186/s13024-021-00426-7.
17. Wojtas, A.M., Sens, J.P., Kang, S.S., Baker, K.E., Berry, T.J., Kurti, A., Daughrity, L., Jansen-West, K.R., Dickson, D.W., Petrucelli, L., et al. (2020). Astrocyte-derived clusterin suppresses amyloid formation in vivo. Molecular neurodegeneration *15*, 71. 10.1186/s13024-020-00416-1.
18. Gregory, J.M., Whiten, D.R., Brown, R.A., Barros, T.P., Kumita, J.R., Yerbury, J.J., Satapathy, S., McDade, K., Smith, C., Luheshi, L.M., et al. (2017). Clusterin protects neurons against intracellular proteotoxicity. Acta Neuropathol Commun *5*, 81. 10.1186/s40478-017-0481-1.
19. Osuka, S., Zhu, D., Zhang, Z., Li, C., Stackhouse, C.T., Sampetrean, O., Olson, J.J., Gillespie, G.Y., Saya, H., Willey, C.D., and Van Meir, E.G. (2021). N-cadherin upregulation mediates adaptive radioresistance in glioblastoma. The Journal of clinical investigation *131*. 10.1172/JCI136098.
