# Supplementary material for: Re-convolving the compositional landscape of primary and recurrent glioblastoma reveals prognostic and targetable tissue states
Source: Nat Commun. 2023 May 4;14:2586. doi: 10.1038/s41467-023-38186-1 (PMC10160047; doi:10.1038/s41467-023-38186-1)
Supplement: Supplementary file 3 — Description of Additional Supplementary Files Document [file 41467_2023_38186_MOESM3_ESM.pdf]

Description of Additional Supplementary Information Files Document

Supplementary Dataset 1-- Single Nucleus and Spatial Transcriptomics Sample Metadata.

Supplementary Dataset 2--Markers of Primary Glioma Subclusters.

Supplementary Dataset 3--Markers of Recurrent Glioma Subclusters.

Supplementary Dataset 4--Markers of Astrocyte Subclusters.

Supplementary Dataset 5--Markers of Myeloid Subclusters.

Supplementary Dataset 6--Differentially Expressed Genes with Cerulenin Treatment in Astrocytes and GBM Explants.

Sheet 1: Differential gene expression comparing astrocytes treated with cerulenin and DMSO. Sheet 2: Differential gene expression comparing IDH-WT GBM explants treated with cerulenin and DMSO. Sheet 3: KEGG Pathway enrichment for differentially expressed genes in cerulenin treated astrocytes.

Supplementary Dataset 7--Gene Signatures associated with Tissue States.

Supplementary Dataset 8--Astrocyte Glioma Co-Culture Experiments.
